# Quantum Dots and Their Multimodal Applications: A Review

**DOI:** 10.3390/ma3042260

**Published:** 2010-03-24

**Authors:** Debasis Bera, Lei Qian, Teng-Kuan Tseng, Paul H. Holloway

**Affiliations:** Department of Materials Science and Engineering, University of Florida, Gainesville, FL, USA; E-Mails: leiqian@ufl.edu (L.Q.); ttkuan@gmail.com (T.K.T.)

**Keywords:** quantum dots, semiconducting nanomaterials, electroluminescence, photoluminescence, solar cells, biological imaging

## Abstract

Semiconducting quantum dots, whose particle sizes are in the nanometer range, have very unusual properties. The quantum dots have band gaps that depend in a complicated fashion upon a number of factors, described in the article. Processing-structure-properties-performance relationships are reviewed for compound semiconducting quantum dots. Various methods for synthesizing these quantum dots are discussed, as well as their resulting properties. Quantum states and confinement of their excitons may shift their optical absorption and emission energies. Such effects are important for tuning their luminescence stimulated by photons (photoluminescence) or electric field (electroluminescence). In this article, decoupling of quantum effects on excitation and emission are described, along with the use of quantum dots as sensitizers in phosphors. In addition, we reviewed the multimodal applications of quantum dots, including in electroluminescence device, solar cell and biological imaging.

## Index

1. Introduction
2. Structure of Quantum Dots
  2.1. Core Structure
    2.1.1. Size *versus* Density of States
    2.1.2. Phases and Phase Transitions
    2.1.3. Doping in Quantum Dots
    2.1.4. Alloying of Quantum Dots
  2.2. Surface Structure
    2.2.1. Surface Passivation
      2.2.1.1. Organically Capped Quantum Dots
      2.2.1.2. Inorganically Passivated Quantum Dots
        2.2.1.2.1. Epitaxial Growth
        2.2.1.2.2. Non-epitaxial Growth
      2.2.1.3. Multi-Shell Structure
    2.2.2. Characterization of Shell Structures
3. Properties
  3.1. Quantum Confinement Effects and Band-Gap
    3.1.1. Effective Mass Approximation Model
    3.1.2. Linear Combination of Atomic Orbital Theory – Molecular Orbital Theory
  3.2. Luminescence Properties
    3.2.1. Radiative Relaxation
      3.2.1.1. Band-Edge Emission
      3.2.1.2. Defect Emission
      3.2.1.3. Activator Emission
    3.2.2. Quantum Yield of Quantum Dots
      3.2.2.1. Reported Quantum Yield
      3.2.2.2. Change of Quantum Yield under Ultraviolet Irradiation
    3.2.3. Non-radiative Process in Quantum Dots
4. Synthesis Processes
  4.1. Top-Down Synthesis Processes
  4.2. Bottom-Up Approach
    4.2.1. Wet-Chemical Methods
      4.2.1.1. Sol-gel Process
      4.2.1.2. Microemulsion Process
      4.2.1.3. Hot-Solution Decomposition Process
      4.2.1.4. Other Synthesis Processes
    4.2.2. Vapor-Phase Methods
5. Application
  5.1. Quantum Dots for Electroluminescence Device Fabrication
  5.2. Downconversion of Blue or Ultraviolet Light
  5.3. Quantum Dots in Solar Cell Device Fabrication
    5.3.1. Quantum Dot Sensitized Solar Cell
    5.3.2. Quantum Dot Dispersed Solar Cell
  5.4. Quantum Dots in Other Optoelectronic Devices
  5.5. Application of Quantum Dots in Bioimaging Applications
    5.5.1. Fluorescence for Bioimaging
    5.5.2. Use of Fluorescence Resonance Energy Transfer in Bioimaging
    5.5.3. Surface Enhanced Raman Spectroscopy
    5.5.4. Radio-Opaque and Paramagnetic Properties
    5.5.5. Magnetic Resonance-based Bioimaging
6. Perspective

## 1. Introduction

Nanostructured materials [1,2,3,4] are of interest because they can bridge the gap between the bulk and molecular levels and leads to entirely new avenues for applications, especially in electronics, optoelectronics and biology. When a solid exhibits a distinct variation of optical and electronic properties with a variation of particle size <100 nm, it can be called a nanostructure, and is categorized as (1) two dimensional, e.g., thin-films or quantum wells, (2) one dimensional, e.g., quantum wires, or (3) zero dimensional or dots. During the last two decades, a great deal of attention has been focused on the optoelectronic properties of nanostructured semiconductors or quantum dots (Qdots) as many fundamental properties are size dependent in the nanometer range. A Qdot is zero dimensional relative to the bulk, and the limited number of electrons results in discrete quantized energies in the density of states (DOS) for nonaggregated zero dimensional structures [5,6]. (Although it is zero dimensional to bulk, it is regarded as a box in quantum mechanics; size of the box is important and discussed later). Sometimes, the presence of one electronic charge in the Qdots repels the addition of another charge and leads to a staircase-like I-V curve and DOS. The step size of the staircase is proportional to the reciprocal of the radius of the Qdots. The boundaries, as to when a material has the properties of bulk, Qdot or atoms, are dependent upon the composition and crystal structure of the compound or elemental solid. An enormous range of fundamental properties can be realized by changing the size at a constant composition and some of these are discusses. Qdots can be broadly categorized into either elemental or compound systems. In this review, we emphasize compound semiconductor-based nanostructured materials and their multimodal applications based on optoelectronic and optical properties.

A process for synthesizing PbS Qdots was developed more than 2000 years ago using low-cost natural materials like PbO, Ca(OH)_2_ and water [7]. The *Romans* and *Greeks* used these materials as cosmetics to dye their hair. In more recent history, control of the size of Qdots in silicate glasses is one of the oldest and most frequently used techniques to control the color of glass. In the early 20^th^ century, CdS and CdSe were incorporated into silicate glasses to get red-yellow colors. In 1932, *Rocksby* [8] used x-ray diffraction (XRD) to determine that precipitates of CdS and CdSe caused the colors. Earlier, semiconductor particles doped glasses were also used in optics as filters. A blue shift of the optical spectrum for nanometer sized CuCl in silicate glass was reported in 1981 by *Ekinov* and *Onushchenko* [9]. In 1982, *Efros* and *Efros* [10] advanced the postulate that quantum size effects (the change of optical and optoelectronic properties with size) could be used to control the color of glass by either changing the size or stoichiometry of CdS_x_Se_1-x_. In 1991, the change in color of colloidal solutions of semiconductor was discussed by *Rosetti* *et al.* [11]. Several different synthesis methods were developed during this period [12,13,14,15]. Over the last two decades, experimental and theoretical research on these nanoparticles has increased significantly [1,16,17,18] in order to explore many basic properties [19,20] of Qdots and attracted by commercialization efforts [21,22]. In this review, we discuss briefly the structure, properties, processing and performance of the Qdots in multimodal applications.

## 2. Structure of Quantum dots

### 2.1. Core Structure

**Figure 1:**
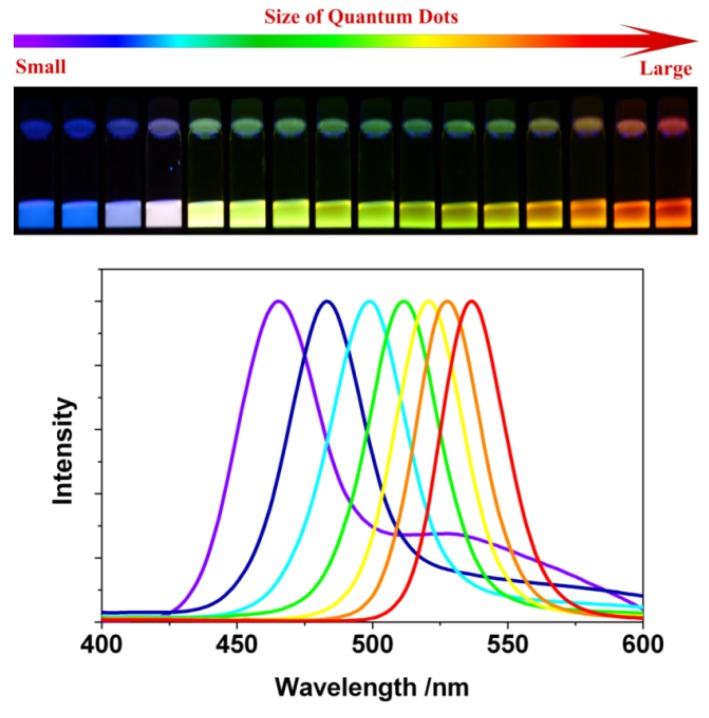
Top: Sixteen emission colors from small (blue) to large (red) CdSe Qdots excited by a near-ultraviolet lamp; size of Qdots can be from ~1 nm to ~10 nm (depends on several parameters, see text for details). Bottom: Photoluminescence spectra of some of the CdSe Qdots [23].

As mentioned before, Qdots have dimensions and numbers of atoms between the atomic-molecular level and bulk material with a band-gap that depends in a complicated fashion upon a number of factors, including the bond type and strength with the nearest neighbors. For isolated atoms, sharp and narrow luminescent emission peaks are observed. However, a nanoparticle, composed of approximately 100*–*10000 atoms, exhibits distinct narrow optical line spectra. This is why, Qdots are often described as artificial atoms (δ-function-like DOS) [18]. A significant amount of current research is aimed at using the unique optical properties of Qdots in devices, such as light emitting devices (LED), solar cells and biological markers. The most fascinating change of Qdots with particle size <~30 nm is the drastic differences in the optical absorption, exciton energies and electron-hole pair recombination. Use of these Qdot properties requires sufficient control during their synthesis, because their intrinsic properties are determined by different factors, such as size, shape, defect, impurities and crystallinity. The dependence on size arises from (1) changes of the surface-to-volume ratio with size, and from (2) quantum confinement effects (discussed later). Nevertheless, Qdots exhibit different color of emission with change in size. Figure 1 shows change of photoluminescence (PL) emission color with size for CdSe Qdots.

#### 2.1.1. Size *versus* Density of States

**Figure 2:**
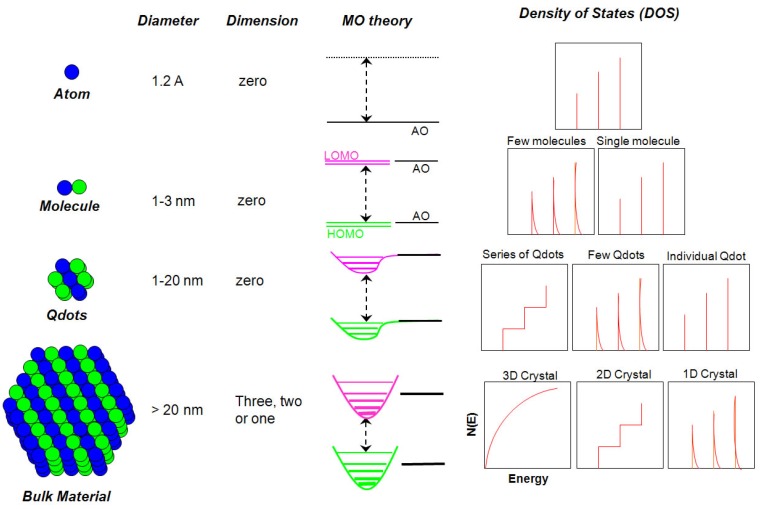
Schematic illustration of the changes of the density of quantum states (DOS) with changes in the number of atoms in materials (MO: molecular orbital; HOMO: highest occupied MO; LUMO: lowest unoccupied MO; AO: atomic orbital) [6,23].

A most unique property of the Qdots is quantum confinement, which modifies the DOS near the band-edges. Schematic diagrams of the DOS as a function of energy in Figure 2 show that Qdots lie between the discrete atomic and continuous bulk materials. Quantum confinement effects are observed when the size is sufficiently small that the energy level spacing of a nanocrystal exceeds kT (where k is *Boltzmann’s* constant and T is temperature). Energy differences > kT restrict the electron and holes mobility in the crystal. Among many properties that exhibit a dependence upon size in Qdots, two are of particular importance. The first is a blue shift (increase) of band-gap energy when the nanoparticle diameters are below a particular value that depends on the type of semiconductor. This is called a quantum confinement effect [24,25] and is discussed below in detail. This effect allows tuning of the energy gap with changes in the Qdot size. The band-gap energy also depends on the composition of the semiconductors as well as the size. The second important property is the observation of discrete, well separated energy states due to the small number of atoms in Qdots compared to the bulk. This leads to the electronic states of each energy level exhibiting wave functions that are more atomic-like. Since the Qdots solutions for *Schrödinger* wave equation are very similar to those for electrons bound to a nucleus, Qdots are called artificial atom, and atomic-like sharp emission peaks are possible. Typical intraband energy level spacings for Qdots are in the range of 10–100 meV. Band-gap can also be tuned by alloying [26,27] the core of the Qdots (discussed below).

#### 2.1.2. Phases and Phase Transitions

II-VI compound semiconductors include the cations of zinc, cadmium and/or mercury combined with anionic oxygen, sulfur, selenium and/or tellurium. These semiconductors generally crystallize in both face-centered cubic (zinc blende) and hexagonal (wurtzite) crystal structures. For example, the equilibrium crystal structures of both ZnO and ZnS are hexagonal, although ZnS often exhibits a metastable cubic or a mixed hexagonal/cubic structures. The II-VI compound semiconductors may exhibit very good luminescence because they have a direct band-gap. In addition, many of the II-VI semiconductors are often used as a host for luminescent activators (discussed later), e.g., ZnS doped with Mn^2+^ which emits yellow light [28]. Near band-edge emission from excitons can be observed from II-VI semiconductors, especially at low temperatures from those materials with a low exciton binding energy.

Qdots exhibits solid-solid phase transition like bulk semiconductors, and these transitions have a substantial influence on the optical properties of Qdots. Phase transitions in bulk materials can be induced by varying pressure, temperature and composition [29,30]. Bulk CdSe may exhibit either a hexagonal wurtzite or a rock salt cubic structure with a direct or indirect band-gap, respectively. Above a pressure of ~ 3 GPa, the CdSe bulk semiconductor can be converted reversibly from low pressure wurtzite to the high pressure rock salt structures [31]. The low intensity optical emission from the rock salt form of CdSe is in the near infrared (NIR) spectral region at 0.67 eV (1.8 μm). Using high pressure XRD and optical absorption, *Tolbert* and *Alivisatos* showed that the wurzite to rock salt structural transformation also occurred in CdSe Qdots [29,30]. The ratio of oscillator strength between direct and indirect structures was unchanged with size of Qdot.

#### 2.1.3. Doping in Quantum Dots

**Figure 3:**
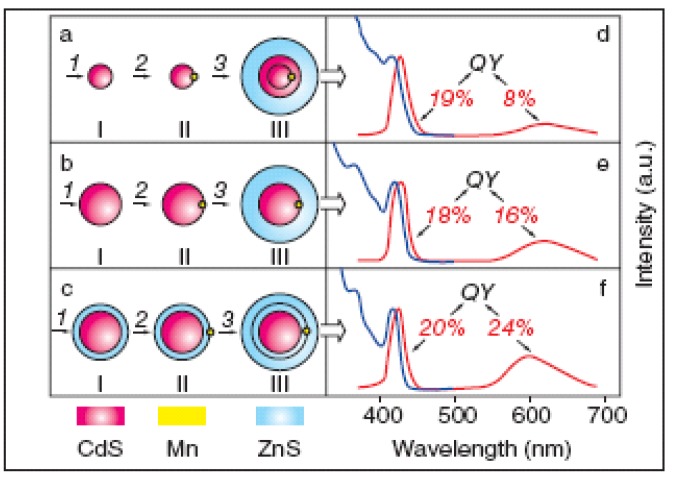
Differences in optical properties of Mn-doped CdS/ZnS core/shell Qdots with different Mn positions; (a) doped in CdS core, (b) at interface of the core/shell structure, and (c) in ZnS shell structure. Core diameter was 3.8 nm and ZnS shell thickness was 1.5 nm. Photoluminescence and photoluminescence excitation spectra are shown in (d), (e) and (f) for IIIa, IIIb and IIIc structure, respectively; (QY: quantum yield). (Reprinted with permission from [47]. Copyright 2006 American Chemical Society).

Doping in Qdots is an important aspect when Qdots are used for various technological applications [32,33,34], especially, optoelectronic, magnetic, biological and spintronic applications. These impurities, called activators, perturb the band structures by creating local quantum states that lies within the band-gaps. In the Qdots, the dopants are found to be auto-ionized without thermal activation due to quantum confinement. When quantum confinement energy (increase of band-gap energy with decreasing size) exceeds *Coulombic* interaction between carrier (hole or electron) and impurity (*n*-type or *p*-type), auto-ionization occurs. Several transition elements such as, Cr [35,36], Mn [35,37,37,38,39], Fe, Co [40], Cu [35,41,42] and Ag [43], and other elements, such as, P [44], B [44], Na [45] and Li [45] were doped in Qdots, for different applications. Optical properties of Qdots can be varied by changing the amounts [46] and the positions (see Figure 3) [47] of dopants in the Qdots. In optoelectronic application of Qdots, doping can play an important role. Conduction in doped Qdot films depends on the size uniformity of Qdots and proximity of neighboring Qdots, so that the orbital overlap among Qdots is maximized. Optical properties of Qdots can be enhanced by doping in Qdots. We discuss more on the optical properties of the doped Qdots in ‘properties of Qdots’ section. Application of doped Qdots can be found mostly in Section 5.5. 

#### 2.1.4. Alloying of Quantum Dots

Keeping the size of a Qdot constant, the band-gap can be engineered by alloying of the core [48,49,50,51,52,53]. Composition of the core materials and/or ratio of alloying materials can change the optoelectronic properties of a Qdot. Such a process has been extensively investigated for the last few years. The process is particularly interesting, because: (1) these nanostructures of semiconductors provide different and nonlinear optoelectronic properties [54] and, (2) Qdot alloyed with multiple semiconductors exhibits a mixed or intermediate optoelectronic properties. NIR emission (600 nm*–*1350 nm) was achieved by synthesizing alloyed CdHgTe Qdots [55] and varying the stoichiometric ratio of two binary semiconductors. In addition, (3) PL emission efficiencies can be improved by minimizing bulk and surface defects [56], and (4) narrow full-width-half-maximum (FWHM) of PL can be achieved. For example, weak quantum confinement regime (explained in ‘structure of Qdots’ section) Zn_x_Cd_1-x_S Qdots were synthesized [57] using a wide band-gap and small *Bohr* radii ZnS and CdS semiconductors. Since these Qdots are in the weak quantum confinement regime (discussed in Section 3.1), inhomogeneous broadening of PL due to size fluctuation is greatly reduced. These Qdots are also being used in many applications including biological imaging [51,58,59]. 

**Table 1:** Change of band-gaps with composition of Zn_x_Cd_1-x_S alloy (Adapted from Reference [54]).

Semiconductor/ Qdot	Value of ‘x’ in Qdot composition	Calculated Particle size Diameter /nm	Band-gap (eV)
ZnS		bulk	3.7
		2.7	4.1
CdS		bulk	2.45
		2.6	2.9
HgS		bulk	~0.0
Zn*_x_*Cd_1-*x*_S	0.14	2.6	3.0
	0.14	3.2	3.0
	0.14	3.5	3.0
	0.14	4.3	2.95
	0.15	2.7	3.0
	0.25	4.0	3.05
	0.34	3.7	3.15
	0.44	4.7	3.5
	0.61	3.9	4.0
Hg*_x_*Cd_1-*x*_S	0.0025	4.0	4.5
	0.005	4.0	4.45
	0.05	4.0	4.4
	0.01	4.0	4.35
	0.2	4.0	3.8
	0.5	4.0	3.25
	0.75	4.0	3.15

### 2.2. Surface Structure

Due to the high surface-to-volume ratio of Qdots, electronic quantum states associated with the surface (called surface states) have significant effects on the optical properties of Qdots. For example, roughly 15% of the atoms in a 5 nm CdS Qdot are at the surface [60]. Such a high surface-to-volume ratio may allow an enhanced or reduced transfer rate of photogenerated charge carriers due to the high density of surface sites. The surface states of the Qdots may influence the optical absorption (photoluminescence excitation – PLE), quantum efficiency, luminescent intensity and spectrum and aging effects [61]. In general, surface states arise from unsatisfied bonds at the reconstructed surface, and may be affected by nonstoichiometry and voids. The energies of these surface states generally lie in the band-gap of the Qdots [62]. Therefore, they can trap charge carriers (electron or hole) and behave as reducing (electron) or oxidizing (hole) agents. These electrochemical reactions or behavior at the surface significantly can affect the overall conductivity and optical properties of Qdots. As a result, surface states have significant effects on the optical and optoelectronic properties of the Qdots. Surface passivation of Qdots can confine the carrier inside the core and improves the optical properties of Qdots. But these passivation layer acts as either insulator or barrier for the conduction of charge.

#### 2.2.1. Surface Passivation

As discussed above, surface defects in Qdots act as temporary ‘traps’ for the electron, hole or excitons, quenching radiative recombination and reducing the quantum yields (QYs). Therefore, capping or passivation of the surface is crucial for development of photostable Qdots. In principle, a perfectly passivated surface of a Qdot has all dangling bonds saturated and, therefore, exhibits no surface state, and all near band-edge states are quantum-confined internally. For a compound semiconductor, if the anion dangling bonds at the surface are not passivated, a band of surface states is expected in the gap just above the valence band-edge. However, passivation of anions with surface cations would also leave dangling bonds that would lead to a broad band of surface states just below the conduction band-edge. Therefore, surface modification of Qdots is very demanding and is generally carried out by depositing an organic or inorganic capping layer on the Qdots.

##### 2.2.1.1. Organically Capped Quantum Dots

**Figure 4:**
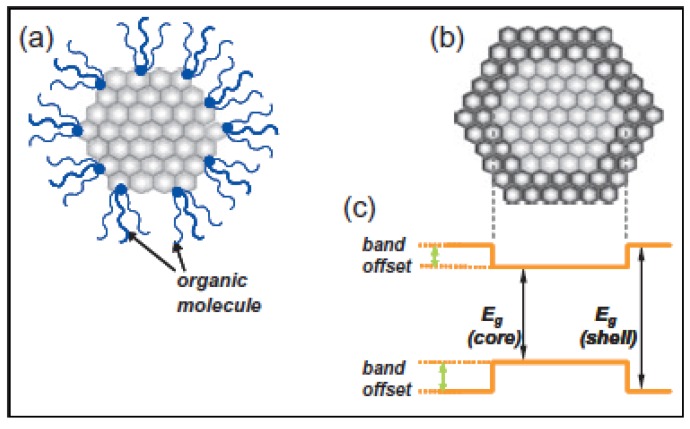
Schematic illustration of (a) an organically capped Qdot and (b) an inorganically passivated Qdot (core/shell structure of Qdot). (c) An energy diagram shows the band-gap difference of core and shell of inorganically passivated Qdots; (E_g_: band-gap) [6].

Generally, monodispersed Qdots are developed by introducing organic molecules that adsorb on the Qdot surface and act as capping agents [63,64,65]. Some advantages of organic capping layers include simultaneous achievements of colloidal suspension and the ability to bio-conjugate the Qdots. However, the selection of organic ligands that bond with surface atoms of the Qdots is a very delicate issue. In general, phosphenes, (e.g., tri-n-octyl phosphene oxide*–*TOPO) or mercaptans (-SH) are the most widely used ligands. Most of the organic capping molecules are distorted in shape and larger than a surface site. As a result, coverage of surface atoms with the organic capping molecules may be sterically hindered. Another crucial issue is the simultaneous passivations of both anionic and cationic surface sites using such capping agents, which is achievable but still complex. Some dangling bonds on the surface are always present when the surface is passivated by organic agents. Finally, the organic capped Qdots are photo-unstable. The bonding at the interface between the capping molecules and surface atoms is generally weak leading to the failure of passivation and creation of new surface states under ultraviolet (UV) irradiation. The surface states of Qdots are known to be sites of preferential photodegradation and luminescence quenching. Figure 4(a) illustrates a Qdot passivated with organic molecules. Chemically reduced bovine serum albumin (BSA) has been used simultaneously to passivate and functionalize the surface of CdTe Qdots to make them water soluble [66]. Denatured BSA (dBSA) conjugated to the CdTe Qdots surface improved the chemical stability and the PL-QY [66]. This study showed that over a pH range of 6 to 9, the solution of dBSA-coated CdTe Qdots were stable and bright, but higher and lower pH values led to dramatic decreases in PL intensity and chemical stability. Similarly, concentrations of dBSA that were too high or too low in the Qdots solution resulted in a decreased PL-QY. Recently, DNA passivated CdS Qdots were reported to be stable in and nontoxic to biological systems [67]. 

##### 2.2.1.2. Inorganically Passivated Quantum Dots

A second approach to passivation of the Qdots surface is the use of inorganic layers, particularly a material with a larger band-gap. The passivating shell is grown either epitaxially (as depicted in Figure 4(b)) or as a non-epitaxial crystalline or amorphous layer on the core. The QY of Qdots is increased by a defect-free, uniform shell coating. When the shell material adapts the lattice parameters of the core during epitaxial growth, coherency strains result and can play an important role in the properties of these core/shell systems. For example, strain may cause the absorption and emission spectra of core/shell Qdots to be red-shifted [68]. The maximum PL efficiency or QY of core/shell Qdots is also dependent upon the thickness of the shell layer. The thickness is less than two monolayers for optimum properties of CdSe/CdS core/shell nanoparticles. Thicker capping layers lead to formation of misfit dislocations which are also nonradiative recombination sites that decrease the PL-QY. More information on shell thickness and PL-QY can be found in section 2.2.2.

###### 2.2.1.2.1. Epitaxial Growth

Generally, a wider band-gap shell material is desired to create a potential barrier around the Qdot core to confine the exciton (see Figure 4(c)). Confinement of the charge carriers into the core region by the band offset potentials result in efficient and photostable luminescence from Qdots. Additional factors to consider when selecting the Qdot inorganic shell material include whether it is hydrophobic or hydrophilic. Most inorganic core/shell Qdots are not compatible with dispersion in water due to the hydrophobic surface property of the shell. For biological application of Qdots, however, an appropriate water-compatible coating, such as an amorphous silica layers, is necessary [69]. For better passivation, the shell material should have a lattice parameter within 12% of the core to encourage epitaxy and minimize strain, and a thickness below the critical value that results in misfit dislocations [70]. The lattice mismatch between CdSe and ZnS (10.6%) is larger than that between CdSe and ZnSe (6.3%) and CdSe and CdS (3.9%), but the band-gap is also larger leading to better exciton confinement. ZnSe is also a good shell material for CdSe, because it has a wider band-gap (2.72 eV) than that of CdSe (1.76 eV). ZnSe also has the same anion (Se) which leads to a larger offset in the conduction bands and therefore to better confinements of the excitons. Luminescence QYs for CdSe/ZnSe core/shell Qdots have been reported in the range of 60–85% [71]. Finally, the smaller lattice mismatch between a CdSe core and a CdS shell facilitates epitaxial growth of a CdS shell. CdSe/CdS core/shell Qdots typically display higher PL-QY with longer PL lifetimes. Our recent investigation shows multi-fold enhancement of CdSe luminescence when amorphous silica layer is introduced onto the CdSe Qdots [72]. Nanocrystalline ZnSe particles have been encapsulated by graphite [73], and enhanced blue emission was observed as compared to unencapsulated ZnSe nanoparticles. Mid-gap defect orange emission was quenched by carbon passivation. 

Although significant improvement was observed by introducing an inorganic shell layer, several evidences on incomplete passivation by the shell are reported in literature. Such an incomplete passivation causes different results like (a) less QY than that from complete passivation [74], (b) a significant amount of permanently dark Qdots [74], (c) photooxidation of core and/or shell [75,76], (d) fluctuation of intensity from core/shell Qdots due to trapping of carriers by the surface states [77].

Inverted core/shell Qdots, e.g., ZnSe/CdSe (with a larger band-gap for the core) Qdots show very interesting optoelectronic properties. They exhibits either type I or type II interfacial band offsets depending on the core radius and the shell thickness [78]. Type I offset is an opposite offset for both the valence and conduction bands. This is the case for bulk ZnSe/CdSe interfaces, where the ZnSe valence band-edge is lower than that in CdSe (energy offset ~0.14eV), while the conduction band-edge is higher (energy offset ~0.86eV). Such an energy alignment results in confinement of both electrons and holes inside the CdSe core which reduces their interactions with surface trap states and improves their QYs. However, the situation can change in the case of nanostructures in which the alignment of quantized energy states is determined not only by bulk energy offsets, but also by the confinement energies determined by the heterostructure dimensions. Core/shell Qdots with type II offsets (valence and conduction band offsets in the same direction) can also provide “spatially indirect” states, in which electrons are spatially confined to the core (or shell) and holes confined to the shell (or core). The emission energy from type II core/shell nanostructures is smaller than the band-gap of either the core or the shell material due to the interfacial energy offsets. Because of the reduced electron-hole wave function overlap, these structures show extended exciton lifetimes and are useful in photovoltaic and photocatalysis applications [79]. With a large red-shift in emission from type-II core/shell Qdots, NIR emission may be possible for *in vivo* bioanalytical and biomedical applications.

###### 2.2.1.2.2. Non-epitaxial Growth

**Figure 5:**
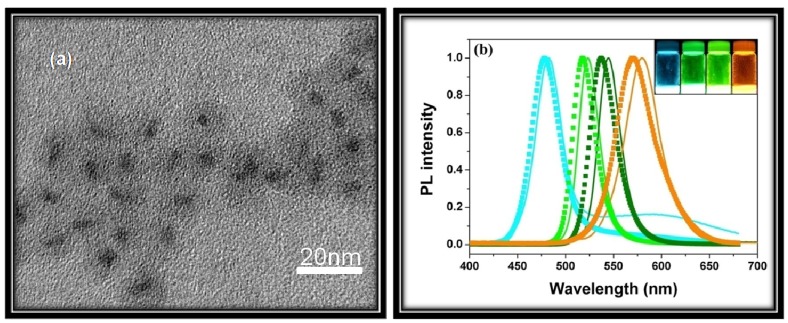
(a) High resolution transmission electron micrograph of silica-coated CdSe Qdots; (b) Photoluminescence (PL) spectra from CdSe (solid) and silica coated CdSe (dotted) Qdots with variation of core size; inset photograph shows emitting color under long wavelength UV-light [23].

As mentioned earlier, Qdots are often synthesized in nonpolar, nonaqueous solvents leaving them hydrophobic. In addition, except for some oxide based Qdots, which are assumed to be less toxic, most of the Qdots contain toxic ions (e.g., cadmium (Cd), selenium (Se) and tellurium (Te). Therefore, an oxide-based coating is important to reduce the toxicity in biological applications. Furthermore, proper functionalization of Qdots is very important for biological applications, as mentioned earlier. To address these issues, a silica shell is grown on the Qdots. A high resolution transmission electron microscopic (HRTEM) image of silica coated CdSe Qdots is shown in Figure 5(a). Aqueous-based synthesis methods generally are used to produce silica-capped Qdots [80]. Recently, it is shown that a silica shell can prevent the leakage of toxic Cd^2+^ from infrared (IR)-emitting CdTe Qdots. Cytotoxicity and the potential interference of Qdots with cellular processes are the subject of intensive studies [81,82]. The silica shell also allows easy functionalization with biomolecules such as proteins [83,84] and results in greater photostability. The luminescent properties of silica-coated Qdots depend on the charge trapped on the surface as well as the local electric field. The field dependent emission from Qdots is called quantum-confined *Stark* effect [85]. External electric field or internal local field results in shifts of both emission wavelength and intensity (shown in Figure 5(b)). By neutralizing a surface positive charge, we recently found that the emission from CdSe Qdots was blue shifted, and the QY increased dramatically. Although there is no attempt found in the literature, the electric field induced change of emission from Qdots can be potentially useful for biological imaging and sensing.

CdS Qdots have also been coated with metal shells resulting in large and fast third-order optical nonlinearity due to surface plasmon resonance (SPR). The collective charge oscillation causes a large resonant enhancement of the local field inside and near the particle which may be used in surface-enhanced *Raman* scattering and in nonlinear optical devices. Qdots coated with a noble metal have been shown to exhibit coupling between the plasmon resonance from the metal and the quantum size effect of the Qdots that give rise to new properties. *Jeang et al.* reported a red-shift of the exciton absorption peak for the Ag/CdS nanocomposite [86]. *Je et al.* investigated the local field-induced optical properties of CdS/Ag core/shell nanocomposites [87]. They confirmed by theoretical calculations that the strong local field created confined *Wannier-Stark* states (*i.e.,* energy spectrum of a crystalline solid in an electric field) that explains the red shift of the exciton peak in the nanocomposite.

##### 2.2.1.3. Multi-Shell Structure

Double shell Qdots are being studied for improved optical properties. As discussed above, the lattice mismatch and differences in band-gap are important to the properties of core/shell Qdots. The band-gaps and band offsets of the core and shell materials are also critical to suppression of tunneling of charge carriers from the core to the surface states of the shell. In the case of CdSe/CdS, the lattice mismatch is small but so are the band offsets. For CdSe/ZnS Qdots, the reverse is true with the lattice mismatch being large along with the band offsets. The advantages of both shell materials are combined in core/shell/shell CdSe/CdS/ZnS Qdots [88,89]. In these double shell nanostructures, the lattice strain at the interface is reduced while large band offsets are maintained. 

Qdots may exhibit significantly low QY due to *Auger* recombination. This process is strongly affected by the confinement. Suppression of non-radiative decay due to *Auger* recombination can be achieved by minimizing wave function overlap of charge carriers [90]. Recently quantum well quantum dots (QWQD) has been introduced to circumvent this issue [90]. In QWQD, a hollow spherical quantum well (QW) surrounds a large band-gap center core Qdot and an outer large band-gap shell passivates the surface, minimizing wave function overlap in the QW [91]. Therefore, higher PL-QY can be achieved from the QWQD compared to the bare Qdots or core/shell Qdots. It is, therefore, of utmost importance to control the structure of QWQD very precisely in order to maximize the QY from Qdots. Multi-shell structured Qdots are also used in biological imaging and investigation in order to achieve bioconjugation [23,92,93,94,95]. 

#### 2.2.2. Characterization of Shell Structures

**Figure 6:**
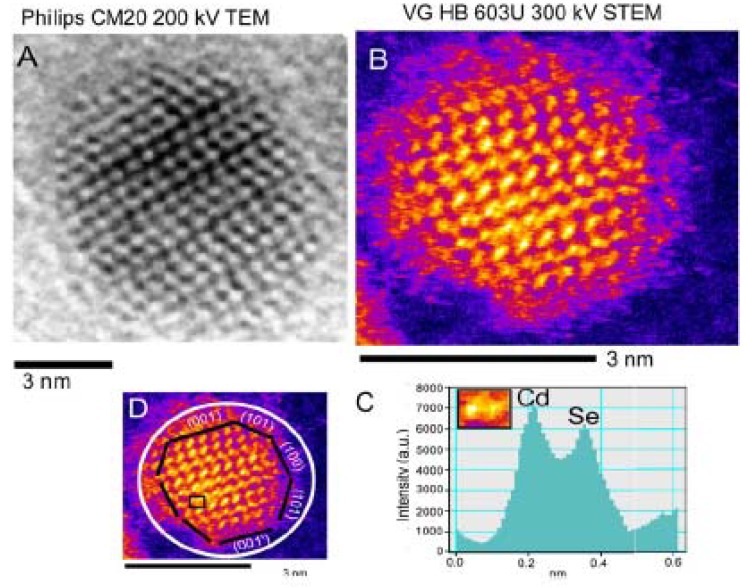
(A) High resolution transmission electron microscopic micrograph *vs.* (B) Z-contrast scanning transmission electron microscopic (STEM) micrograph of a CdSe Qdots. (C) STEM image shows mass distribution to determine a crystallite facet; (D) CdSe crystal facets are shown in STEM micrograph [reprinted from [98] with permission from Elsevier].

Recently, chemical distribution of shell materials on the CdSe/ZnS core/shell Qdots was studied by *Yu et al.* [96] by using scanning transmission electron microscopy (STEM) coupled with electron energy loss spectroscopy (EELS). According to their analyses on electron EELS spectra and simultaneous annular dark field (ADF) signal, the ZnS shell was well-defined around the CdSe core. However, the distribution of shell material was highly anisotropic possibly due to differences in chemical activity of the crystal faces of the core CdSe [97]. Accuracy of the measurement is questionable as collection of the localized EELS spectra was carried out from a subnanometer area on a single Qdot which can be easily overwhelmed by either near-by areas of same Qdots or neighbor Qdots due to movement of Qdots under a high-energy electron beam. Z-contrast STEM analysis possesses several advantages over conventional transmission electron microscopy (TEM) [98]. Figure 6 shows a Z-contrast STEM *versus* a high resolution TEM or HRTEM micrograph of a CdSe Qdot. In Z-contrast STEM, incoherently scattered electrons are collected by a high-angle annular dark field (HAADF) detector. According to the *Rutherford* scattering equation, the intensity of scattered electrons is proportional to square of the atomic number for sufficiently high angular range. Therefore, mass-contrast can be observed directly from images. Although *McBride et al.* [99,100] demonstrated the imaging of both bare CdSe Qdots and core/shell Qdots by using an aberration-corrected Z-contrast STEM, the real challenge is to interpret bonding information from TEM analysis. In addition, Z-contrast STEM images of Qdots with an amorphous shell, e.g., silica, is near-impossible. 

X-ray photoelectron spectroscopy (XPS) is a critical tool to analyze the surface of Qdots [101,102]. One of the earliest and most extensive XPS studies on the nature of the CdSe Qdots was carried out by *Katari et al.* [101]. They showed that the XPS core level positions for Cd and Se from covalently bound monodispersed CdSe to Au on Si substrate were in agreement with those of bulk CdSe. However, both Cd and Se were oxidized in the case of completely washed and air-exposed CdSe Qdots [101]. XPS was also used to verify the shelling in core/shell Qdots [76,103,104]. All abovementioned studies demonstrated that composition of surface ligand and shell coverage, and surface oxidation of Qdots can be analyzed by XPS. However, XPS cannot be used to analyze specifically surface, shell structure and core-shell interface bonding of Qdots, because the escape length in conventional XPS is comparable to the size of the Qdots. In addition, the smallest spot size in XPS analysis is much larger (~1 µm) than the Qdots.

As discussed in previous sections, thickness of the shell has profound effects on luminescence properties of Qdots. For example, a single monolayer of surface passivating inorganic shell on a Qdot increases the QY by a factor of 3 [70]. However thick-shell on Qdots can reduce [70] the QY of the Qdot significantly by formation of misfit dislocations which are also nonradiative recombination sites that decrease the QY. An optimum thickness is important in order to maximize the PL-QY. Actual thickness of the shell of a core/shell Qdot is difficult to measure as (1) thickness is very small compared to core to be observed in TEM, and (2) it may be epitaxially grown so that could not be resolved in XRD studies. Traditional characterization techniques, such as, TEM, XRD, and XPS confirms the addition of a few monolayers onto Qdots. Currently, thickness of the shell of a core/shell system is approximated either by comparing TEM micrographs of bare and core/shell Qdots or by calculating differences from average particle sizes of core and core/shell Qdots using XRD [97]. However, these characterization techniques do not provide any information about interface bonding of core and shell. This warrants the need of new characterization techniques that can provide more complete information on the shell structure in the core/shell Qdots and QWQD. 

## 3. Properties

### 3.1. Quantum Confinement Effects and Band-Gap

Quantum confinement generally results in a widening of the band-gap with a decrease in the size of the Qdots. The band-gap in a material is the energy required to create an electron and a hole at rest (*i.e.,* with zero kinetic energy) at a distance far enough apart that their *Coulombic attraction* is negligible. If one carrier approaches the other, they may form a bound electron-hole pair, *i.e.,* an exciton, whose energy is a few meV lower than the band-gap. This exciton behaves like a hydrogen atom, except that a hole, not a proton, forms the nucleus. Obviously, the mass of a hole is much smaller than that of a proton, which affects the solutions to the *Schrödinger* wave equation. The distance between the electron and hole is called the exciton* Bohr* radius (r_B_). If m_e_ and m_h_ are the effective masses of electrons and holes, respectively, the exciton *Bohr* radius for bulk semiconductor can be expressed by Equation 1, where ε, ћ, and e are the optical dielectric constant, reduced *Planck’s* constant and the charge of an electron, respectively.

(1) $$r_{B}=\frac{\hbar^{2}\varepsilon }{e^{2}}\left(\frac{1}{m_{e}}+\frac{1}{m_{h}}\right)$$

If the radius (R) of a Qdot approaches r_B_, *i.e.,* *R ≈ r_B, _*or *R < r_B_*_, _the motion of the electrons and holes are confined spatially to dimension of the Qdot which causes an increase of the excitonic transition energy and the observed blue shift in the Qdot band-gap and luminescence. The exciton *Bohr* radius is a threshold value, and the confinement effect becomes important when the Qdot radius is smaller. For small Qdots, the exciton binding energy and biexciton binding energy (exciton-exciton interaction energy) is much larger that for bulk materials [105]. Note that for a material with a relatively higher ε or smaller m_e_ and m_h_, the r_B_ is larger. Two detailed theoretical approaches are used to better predict the exciton properties, specifically the effective mass approximation (EMA) model and linear combination of atomic orbital (LCAO) theory. Below, we discuss these two theories in brief.

#### 3.1.1. Effective Mass Approximation Model

This approach, based on the ‘Particle-in-Box Model’, is the most widely used model to predict quantum confinement. It was first proposed by *Efros* and *Efros* [10] in 1982 and later modified by *Brus* [106]. It assumes a particle in a potential well with an infinite potential barrier at the particle boundary. For a particle free to assume any position in the box the relationship between its energy (E) and wave vector (k) is given by Equation 2.

(2) $$E=\frac{\hbar^{2}k^{2}}{2m^{*}}$$

In the EMA model, this relationship (Equation 2) is assumed to hold for an electron or hole in the semiconductor, therefore the energy band is parabolic near the band-edge. The shift of band-gap energy (ΔE_g_) due to confinement of the exciton in a Qdot with a diameter R can be expressed as follows (Equation 3), where, μ is the reduced mass of an electron-hole pair and E^*^_Ry _is *Rydberg* energy.

(3) $$\Delta E_{g}=\frac{\hbar^{2}\pi^{2}}{2\mu R^{2}}-\frac{1.8e^{2}}{\varepsilon R}=\frac{\hbar^{2}\pi^{2}}{2R^{2}}\left(\frac{1}{m_{e}}+\frac{1}{m_{h}}\right)-\frac{1.78e^{2}}{\varepsilon R}-0.248E_{Ry}^{*}$$

**Figure 7:**
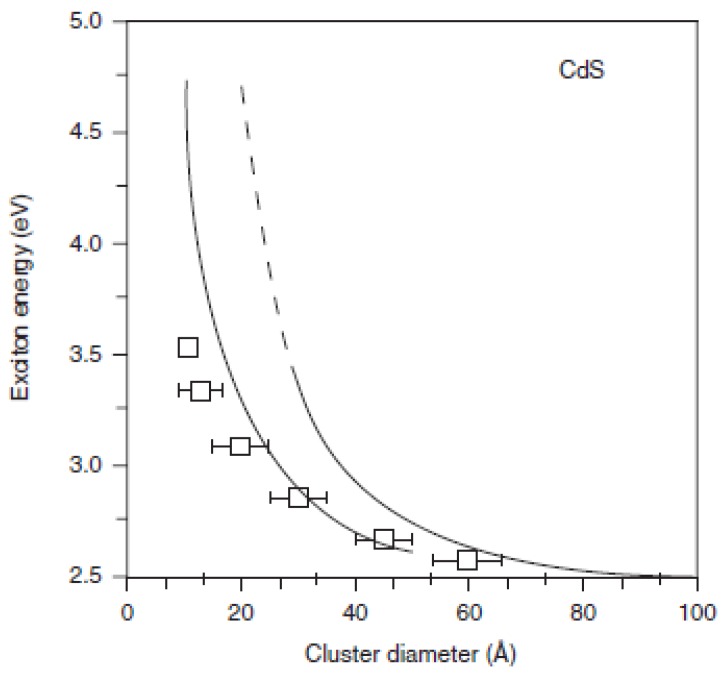
Experimentally and theoretically determined band-gap as a function size of CdS Qdots. Broken line: calculated parameters based on effective mass approximation, solid-line: tight-bonding calculation; squares: experimental data (Reprinted with permission from [60]. Copyright 1991 American Chemical Society).

The first term of the Equation 3 represents a relation between ‘particle-in-a-box’ quantum localization energy or confinement energy and the radius of the Qdot (R), whereas the second term shows the *Columbic* interaction energy with a R^-1^ dependence. The *Rydberg* energy term is size independent and is usually negligible, except for semiconductors with small dielectric constant [60]. Based on Equation 3, the first excitonic transition (*i.e.,* the band-gap) increases as the Qdot radius (R) decreases (quantum localization term shifts to higher energy with lower R value (R^-2^) and *Columbic* terms shifts excited electronic state to lower value (R^-1^)). However, the EMA model breaks down in the small Qdot regime [14,60] because the *E-k* relationship can no longer be approximated as parabolic. Figure 7 shows such a deviation of theoretically predicted band-gaps for CdS Qdots from the experimental values.

#### 3.1.2. Linear Combination of Atomic Orbital Theory*–*Molecular Orbital Theory

**Figure 8:**
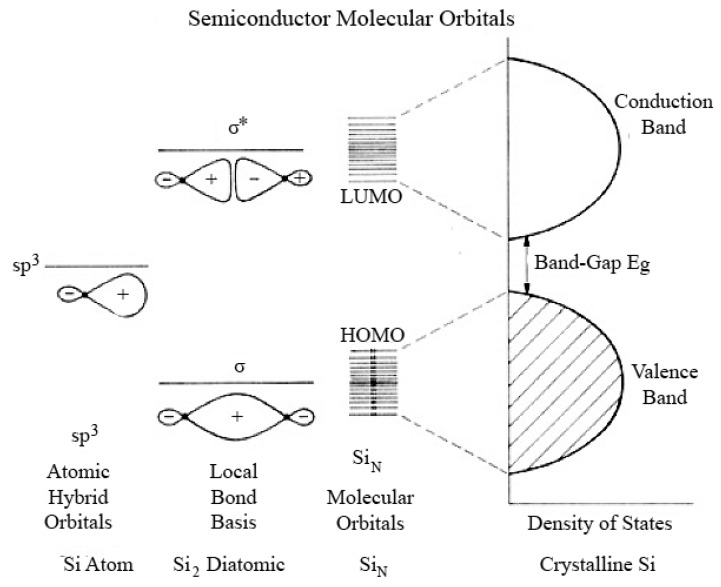
Combination of atomic orbital to molecular orbital and to band-gap of silicon molecule (Reprinted with permission from [107]. Copyright 1990 American Chemical Society).

A model based on a linear combination of atomic orbitals*–*molecular orbitals (LCAO-MO) provides a more detailed basis for predicting the evolution of the electronic structure of clusters from atoms and/or molecules to Qdots to bulk materials, and predicting the dependence of band-gap on size of the crystals. Figure 8 shows the results of this approach pictorially. In a diatomic Si molecule, the atomic orbitals (AO) of two individual atoms are combined, producing bonding and anti-bonding molecular orbitals. In this approach, nanosized Qdots are considered as large molecules. As the number of atoms increase, the discrete energy band structure change from large steps to small energy steps, *i.e.,* to a more continuous energy band. The occupied (bonding) molecular orbital quantum states (equivalent to the valence band) are called the highest occupied molecular orbital (HOMO) levels. The unoccupied antibonding orbitals (equivalent to the conduction band) are called the lowest unoccupied molecular orbital (LUMO) levels. The energy difference between the top of the HOMO and bottom of the LUMO (equal to the band-gap) increases and the bands split into discrete energy levels reduced mixing of AOs for a small number of atoms. Therefore, the small size of the Qdots results in quantized electronic band structures intermediate between the atomic/molecular and bulk crystalline MOs.

Compared to the effective mass approximation, the LCAO-MO model provides a methodology to calculate the electronic structure of much smaller Qdots. In contrast, this method cannot be used to calculate the energy levels of large Qdots due to mathematical complexity and limitations of the computing systems. Nevertheless, the degree of quantum confinement is determined by the ratio of the radius of a Qdot (*R*) to bulk excitonic *Bohr* radius (r_B_). At crystal sizes greater than the excitonic *Bohr* diameter (2r_B_), semiconductor crystals exhibit translational motion confinement of the fully coupled exciton due to a strong *Coulombic* interaction between the electron and holes, *i.e.,* exhibits single-particle confinement behavior (sometimes called the strong confinement regime). In the intermediate size range (*R ≤ r_B_*), the transition energies of photoexcited carriers in the crystal are determined by the relative strengths of the kinetic energy of confinement and the electron-hole interaction. 

**Figure 9:**
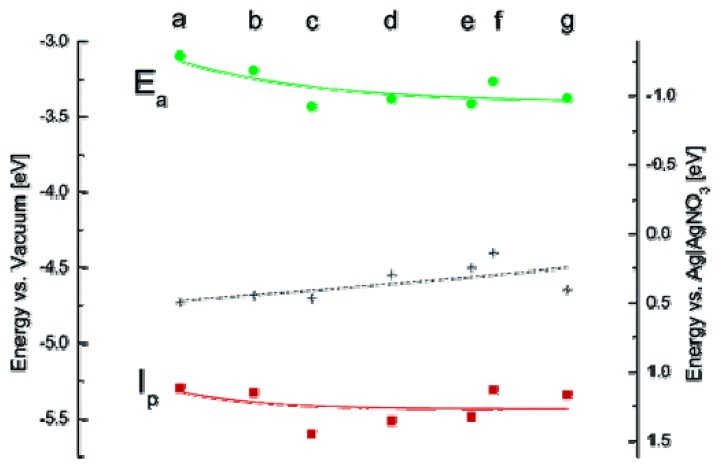
Experimental values of ionization potential (I_p_) and electron affinity (E_a_) of different sized TOPO coated CdSe Qdots (a: 2.3 nm, b: 3.1 nm, c: 3.2 nm, d: 3.5 nm, e: 3.7 nm, f: 3.8 nm, g: 4.0 nm) (Reprinted with permission from [62]. Copyright 2005 American Chemical Society).

Band-gap of Qdots can be determined by electrochemical measurement using Qdots films. Cyclic voltametry (CV) are often employed [108,109,110,111,112,113] to determine the oxidation and reduction potential of the film of Qdots to be measured using a standard three-electrode cell. CV is a dynamic electrochemical method in which current-potential curves are traced at a pre-defined scan rates. Qdots-coated gold plate, platinum wire or indium tin oxide film on glass substrates are often used as working electrodes and platinum electrode acts as a counter electrode. The cell potential is generally normalized to reference electrode using Fc/Fc^+^ couple. Band-gaps of CdS [109], CdSe [110,113], CdTe [111,112], and Qdots are determined using CV. Figure 9 shows calculated ionization potentials of different sized CdSe Qdots. 

### 3.2. Luminescence Properties

After excited by an external energy, e.g., photon for photoluminescence, electric field for electroluminescence, primary electron for cathodoluminescence *etc.*, electron and hole possess high energies due to transitions of electron from ground state to an excited state. The energies associated with such optical absorptions are directly determined by the electronic structure of the material. The excited electron and hole may form an exciton, as discussed above. The electron may recombine with the hole and relax to a lower energy state, ultimately reaching the ground state. The excess energy resulting from recombination and relaxation may be either radiative (emits photon) or nonradiative (emits phonons or *Auger* electrons). Some radiative events from band-edge, defects and nonradiative processes are discussed in brief.

#### 3.2.1. Radiative Relaxation

Radiative relaxation results in spontaneous luminescence from Qdots. Such luminescence may result from band-edge or near band-edge transitions or from defect and/or activator quantum states. We discuss such emissions in the following sections.

##### 3.2.1.1. Band-Edge Emission

The most common radiative relaxation processes in intrinsic semiconductors and insulators are band-edge and near band-edge (exciton) emission. The recombination of an excited electron in the conduction band with a hole in the valence band is called band-edge emission. As noted above, an electron and hole may be bound by a few meV to form an exciton. Therefore, radiative recombination of an exciton leads to near band-edge emission at energies slightly lower than the band-gap. The lowest energy states in Qdots are referred as *1s_e_-1s_h_* (also called exciton state). The full width at half maximum (FWHM) of a room-temperature band-edge emission peak from Qdots varies from 15 to 30 nm depending on the average size of particles. For ZnSe Qdots, however, the luminescence can be tuned by size over the spectral range 390*–*440 nm with FWHM as narrow as 12.7–16.9 nm [114,115]. The optical absorption spectrum reflects the band structure of the materials. While PL from bulk semiconductors is fairly simple and well-understood, and can be explained by parabolic band theory, the PL from Qdots raises several questions. For example, radiative lifetime of 3.2 nm sized CdSe Qdots can be 1 μs at 10K compared to bulk (~1 ns) [116,117]. This was explained by the fact that there were surface states that involved in emission [116]. Band structures of semiconductors are often determined from either absorption spectra or PLE spectra. The study [116] also showed that these two spectra exhibited different characteristics when these spectra were acquired at 15 K. The PLE spectrum was associated with couple of additional peaks along with *1s_e_-1s_h_*. *Bawendi et al.* assigned these peaks as formally forbidden *1s_e_-1p_h_* and *1s_e_-2s_h_*. It was also observed experimentally that the *Stokes* shift was size dependent. For a large size CdSe Qdots (5.6 nm), the *Stokes* shift was found to be 2 meV whereas for a same Qdots of size 1.7nm, the value could be 20 meV. Such a discrepancy was explained, theoretically and experimentally, in terms of increase of distance between optically active state and optically forbidden ground exciton state with decreasing the size of Qdots [117,118]. 

**Figure 10:**
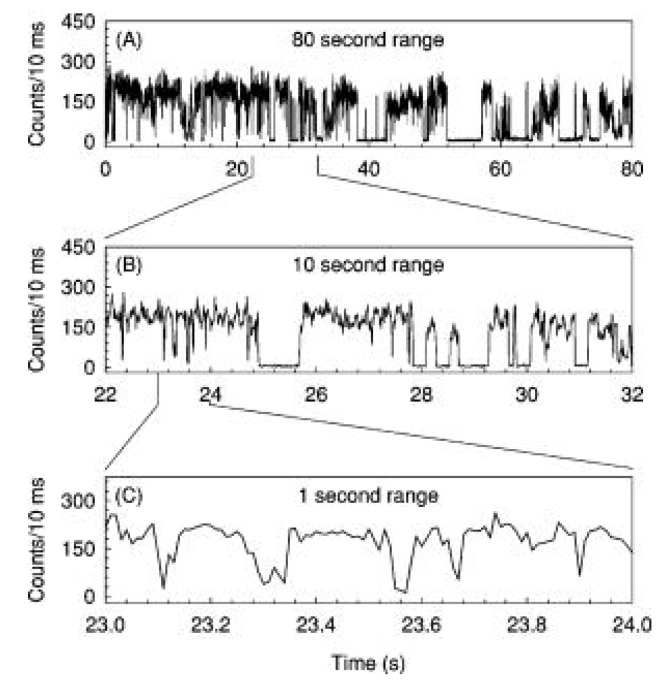
Blinking effect during luminescence from a single 2.9 nm sized CdSe Qdot (reprinted with permission from [121]. Copyright 2000, American Institute of Physics).

As mentioned in previous sections, Qdots have a number of advantages over organic dyes in bio-applications, e.g., better photostability, wide absorption edges, and narrow, tunable emission. However, they may exhibit a random, intermittent luminescence which is called ‘blinking’. In blinking, a Qdot emits lights for a time followed by a dark period (shown in Figure 10). In 1996, *Nirmal et al.* [77] observed this switching between an emitting and a non-emitting state from a single CdSe Qdot at room temperature. The postulated mechanism of blinking was a photoinduced ionization process [119] which leads to a charged Qdots that results in a separation between electrons and holes. Based on this model, the Qdots would be dark for the lifetime of the ionized state. Nonradiative *Auger* recombination process would be expected to dominate the quenching of ionized Qdots [119]. However, the experimental results don’t completely support this model. For example, a photo-induced *Auger* process should exhibit a quadratic dependence of the average blinking time on excitation intensity, whereas the experimental result showed a linear behavior. In addition, the bright and dark periods followed an inverse power law [120] given by Equation 4, where, P(t) is a probability of the blinking period, m is an exponent between 1 and 2 and A is a constant.

(4) $$P(t)=A.t^{-m}$$

Several additional mechanisms have been proposed to explain the blinking [121,122,123,124], including thermally activated ionization, electron tunneling through fluctuating barriers or into a uniform distribution of traps, or resonant electron tunneling between the excited states of Qdots and dark-trap states that wander randomly in energy. Despite tremendous efforts, the blinking effect is still not properly explained.

##### 3.2.1.2. Defect Emission

Radiative emission from Qdots also comes from localized impurity and/or activator quantum states in the band-gap. Defect states lie inside the bands themselves [124]. Depending on the type of defect or impurity, the state can act as a donor (has excess electrons) or an acceptor (has a deficit of electrons). Electrons or holes are attracted to these sites of deficient or excess local charge due to *Coulombic* attraction*.* Similar to the case of excitons, trapped charge on defect/impurity sites can be modeled as a hydrogenic system, where binding energy is reduced by the dielectric constant of the material [125]. These defects states can be categorized into either shallow or deep levels, where shallow level defect states have energies near the conduction band or valence band-edge. In most cases, shallow defect exhibits radiative relaxation at temperatures sufficiently low so that thermal energies (kT) do not excite the carriers out of the defects or traps states. Deep levels, on the other hand, are so long-lived that they typically experience nonradiative recombination.

Luminescence from these defect levels can be used to identify their energy and their concentration is proportional to the intensity. Both PL spectral distribution and intensity change with changes of the excitation energy due to contributions from different defect energy levels and the band structure of the host. The excitation energy also determines the initial photoexcited states in the sample, but this state is short-lived because of thermalization of the photoexcited carriers *via* phonon emission, as discussed above. Relaxation to within kT of the lowest vibrational level of the excited states is usually orders of magnitude faster than the recombination event [125].

**Figure 11:**
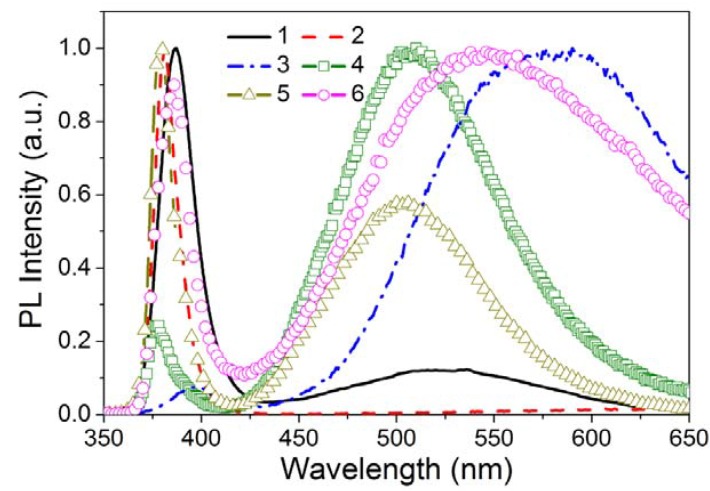
Room temperature PL spectra of various ZnO nanostructures: (1) tetrapods, (2) needles, (3) nanorods, (4) shells, (5) highly faceted rods, and (6) ribbons/combs, [reproduced with permission from [128]. Copyright Wiley-VCH Verlag GmbH & Co. KGaA].

Defect states are expected at the surface of a Qdot despite the use of various passivation methods, because of the large surface-to-volume ratio, discussed above. The concentration of surface states on the Qdots is a function of the synthesis and passivation processes. These surface states act as traps for charge carriers and excitons, which generally degrade the optical and electrical properties by increasing the rate of nonradiative recombination. However, in some cases, the surface states can also lead to radiative transitions, such as in the case of ZnO nanostructures (Figure 11). Powders of ZnO have a green emission from defects along with a band-edge near UV emission (the band-gap of ZnO is 3.37 eV or 386 nm) at room temperature [126,127,128,129,130]. It is also reported that the green emission suppressed the band-edge emission. Theoretical and experimental studies [131,132] showed that the defect states in a ZnO Qdot can be of several types including neutral, singly or doubly charged Zn vacancies (V_Zn_), neutral or singly charged oxygen vacancies (V_O_), singly charged or neutral interstitial Zn (Zn_i_), interstitial O (O_i_), a complex of V_O_ and Zn_i_ (V_O_Zn_i_), a complex of V_Zn_ and Zn_i_ (V_Zn_Zn_i_), and substitution O at Zn position (O_Zn_). According to *Aleksandra et al.* [132], the singly charged oxygen vacancy (V_O+_) is located at 2.28 eV below the conduction band in the ZnO band-gap and results in an emission at ~ 540 nm. The most widely, but not universally, accepted mechanism for green luminescence from ZnO is the electron-hole recombination on singly ionized oxygen vacancies. In solution-based synthesis, the oxygen vacancies appear to be intrinsic and may result from heterogeneous nucleation and growth, enhanced by the large surface area. If the radiative center is associated in part with the surface, their concentration would be expected to decrease with aggregation of Qdots as observed [133].

##### 3.2.1.3. Activator Emission

Luminescence from intentionally incorporated impurities is called extrinsic luminescence. The predominant radiative mechanism in extrinsic luminescence is electron-hole recombination which can occur *via* transitions from conduction band to acceptor state, donor state to valance band or donor state to acceptor state. In some cases, this mechanism is localized on the activator atom center. In some many cases, the selection rule is relaxed due to mixing of orbitals, such as *d-p* mixing in a crystal or ligand field where the orbitals are split into hyperfine structures. Therefore, *d-d* transition is allowed in some cases for transition elements. For Mn^2+^, the lifetime of the luminescence [76] is in the order of millisecond due to the forbidden *d-d* transition. Similarly, *f-f* transition are also often observed for rare earth elements (e.g., Tm^3+^, Er^3+^, Tb^3+^,and Eu^3+^), although the *f* levels are largely unaffected by the crystal field of the host due to shielding by the outer *s-* and *p-*orbitals [134]. Due this shielding effect, *f-f* transitions typically have atomic-like sharp peaks in the emission spectra.

The optical properties of doped ZnO Qdots have also been widely investigated [135,136,137]. Doping with Er or Mn has been reported to result in preferential orientation of nanorods perpendicular to the substrate [138]. ZnO Qdots have also been doped with rare-earth elements, such as Tb [139], Ce [140], Eu [141] and Dy [142]. In the case of Tb-doped ZnO Qdots, emissions from both Tb and defect states were observed. The emission from Tb was found to increase with increasing Tb concentrations, while that from defect states decreased. Eu-related emission was observed from ZnO:Eu nanorods for a suitable excitation wavelength. However, Dy-doped ZnO nanowires exhibited a relatively strong UV emission with a very weak emission from Dy. The effects of doping Mn in ZnO nanoparticles depend strongly on the synthesis conditions [143]. The Mn was found to quench green emission [143], while others reported either a reduction in both UV and defect emissions [144] or a blue shift and increase in UV peak intensity [145]. Very similar spectra from ZnO and Mn-doped ZnO were observed after annealing at 800 °C [146]. Other dopants, such as sulfur and copper, have been studied in ZnO Qdots. Increased intensity and changes in spectral distribution of the broad green defect emission with S doping has been reported [147,148]. 

**Figure 12:**
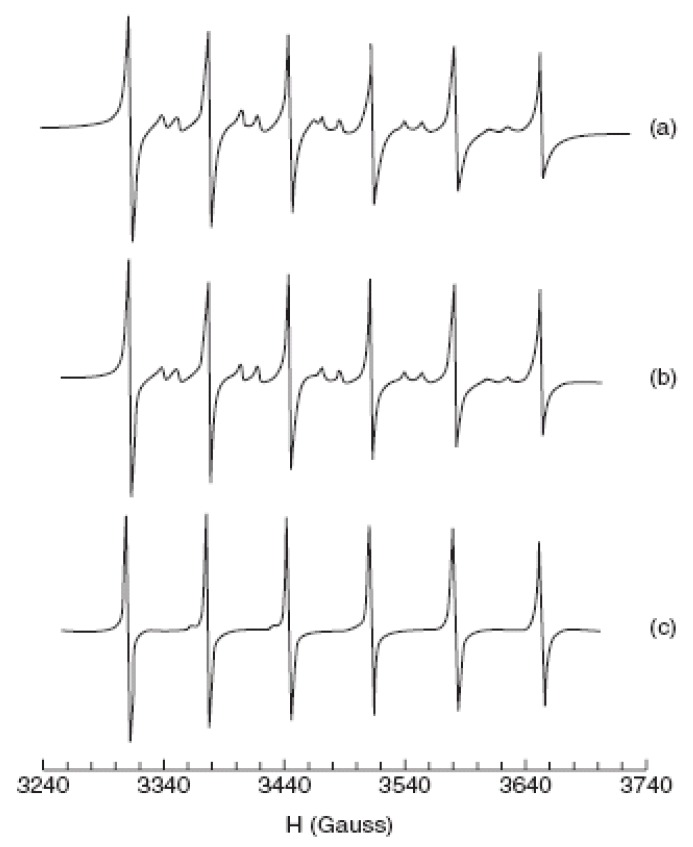
EPR spectra for Mn^2+^in ZnS:Mn sample measured at room temperature; (a), 0.003% Mn^2+^(experimental), (b) 0.008% Mn^2+^(experimental), and (c) 0.008% Mn^2+^ (simulated) (reprinted with Permission from [156]. Copyright 2004 American Chemical Society).

Doped ZnS Qdots [149,150] are very important semiconductor nanomaterials, with Mn^2+^-doped ZnS Qdots being one of those most studied as a phosphor [151]. In 1994, *Bhagrava* *et al.* [152] reported very high PL-QY (~18%) from ZnS:Mn Qdots. Coincident with the intensity enhancement, they reported shorter luminescent lifetimes for the Mn^2+^ emission (decrease from hundreds of microseconds for the bulk to nanoseconds in nanocrystals) [153,154]. The increased intensity was attributed to an efficient energy transfer from the ZnS host to Mn^2+^ ions facilitated by mixed electronic states. Hybridization of atomic orbitals of ZnS and *d*-orbitals of Mn^2+^ in the nanoparticles was suggested to also be responsible for the relaxation of selection rules for the spin-forbidden *^4^T_1_→^6^A_1_* transition of Mn^2+^, leading to the short emission lifetimes. Subsequent research demonstrated that while the QY of passivated ZnS:Mn Qdots could be high, the luminescent lifetimes were not significantly smaller from those of the bulk material. The luminescence properties were, however, found to be dependent upon the S^2-^ and Mn^2+^ concentrations, as well as, the structural properties of the Qdots. The *^4^T_1_→^6^A_1_* Mn^2+^ emission intensity generally increases with increasing doping Mn^2+^ concentration [155] and a quenching of Mn^2+^ emission was observed at high Mn^2+^ concentrations (>0.12 at %). The local environment around the Mn^2+^ in the Qdots has been studied using X-ray absorption fine structure (XAFS) and electron spin resonance (ESR). XAFS data showed that the Mn^2+^ substituted on the tetrahedral Zn^2+^ site in the lattice. ESR data were consistent with this conclusion, showing a spectrum for Mn^2+^ spins typical of a tetrahedral crystal field [156]. In some cases for ZnS:Mn Qdots, the ESR spectra show a Mn^2+^ signal with octahedral symmetry (See Figure 12), but the location of this defect site is not fully understood. It has also been suggested [156] that this signal resulted from Mn^2+^ on the surface of the Qdots *versus* in the interior, but this assignment has been disputed and attributed to Mn-Mn clustering at high concentrations.

#### 3.2.2. Quantum Yield of Quantum Dots 

The accurate quantum yield or QY measurements are crucial for Qdots. It is commonly noted that the values of the QYs do not agree among several reports. This may be due to one of the following reasons or a combination thereof: (1) different approaches to measuring QYs, (2) inappropriate concentrations or optical density (OD) of sample or standards, where *Beer’s* law does not follow or concentration quenching occurs, (3) change of slits between samples and standards during measurements; (4) use of different excitation wavelength for PLE or first absorption peak, (5) no overlap between emission wavelengths of samples and standards, and/or (6) instrumental error/s, such as, wavelength shift, instability of source light *etc.* It is also known that inorganic semiconducting Qdots do not behave like fluorescent molecules, such as dyes. In previous, we have used the equation provided by IUPAC [157] for determining QY of Qdots [69,94,97,102,158,159]. The procedure to determine the QY is by comparing the integrated emission intensity from the Qdots to that from standards. The optical densities of the Qdots and the standard/s are determined. The absorbance value for both samples and standards should be kept below 0.08 at the excitation wavelength. In previous, we used the following equation to measure QY [102,159]. 

(5) $$QY=QY_{St}\frac{1-10^{-A_{St}}}{1-10^{-A}}\times \frac{\eta^{2}}{\eta_{St}^{2}}\times \frac{I}{I_{St}}$$

In Equation 5, QY and QY_St_ are quantum yields (St: Standards), A and A_St_ are absorbance values at the excitation wavelength, η and η_St_ are refractive indices of the solvents, and I and I_St_ are integrated emission areas for the Qdots samples and the standards, respectively. Same excitation wavelength should be used for both the sample and standards.

##### 3.2.2.1. Reported Quantum Yield

In this section, we review some of the QY values that are reported in literature. In the following table (Table 2), we summarized some of the research reports from literatures.

**Table 2:** Some of the literature reported Qdot Quantum Yield data for selected systems.

Quantum Dot	Size (nm)	Emission	Quantum Yield	Standard (QY)	Specification	Ref
CdSe/ZnS	2.7–3	Excitation 470 nm Emission range: 480–850 nm	50%	Rhodamine 560 in ethanol	Shell thickness: ~0.6nm	[103]
CdSe	4.2		20%		Bare	[104]
CdSe/ZnS	4.2		50%		1.5 monolayer of ZnS	[104]
CdSe/CdS	2.3 3.0		59% 84%		2.1 MLr CdS 1.8 MLr CdS	[70]
ZnSe	4.3–6	360–420 nm	20–50%			[160]
CdSe/ZnS	2.0 2.6 4.6 5.6	O.D. 0.1	36% 49% 30% 27%	Rhodamine 590, 610, 640		[161]
CdSe/ZnS	3.7		66%		1.6 MLr ZnS	[162]
ZnSe:Mn	2.7–6		22% at RT 75% at 50K			[163]
CdSe	7.5	O.D. 0.1	85%	Coumarin 540 (62% @ 458nm), Rhodamin 6G (95% @ 528nm), 3B (50% @ 550nm), 640 (100% @ 570 nm), LD 690 (63% @616 nm)	As synthesized	[164]
CdSe	7.5	O.D. 0.1	85%	Coumarin 540 (62% @ 458nm), Rhodamin 6G (95% @ 528nm), 3B (50% @ 550nm), 640 (100% @ 570 nm), LD 690 (63% @616 nm)	As synthesized	[164]
PbSe	4–5		6–20%			[165]
CdSe	2–8	excitation: 400 nm; OD: ≤ 0.1)	50–80%	Rhodamin B in ethanol (90% @ 400 nm)		[166]
CdSe CdSe/CdS CdSe/CdS/ZnCdS CdSe/CdS/ZnCdS/ZnS	3.8 5.2 7.6 8.9		30% 60% 65% 80%		Bare CdS: 2ML ZnCdS: 2ML ZnS: 2.5 ML	[89]
CdSe/ZnS ZnSe/CdSe/ZnS			90% (550–650 nm) 70% (510–560 nm)			[167]
CdSe CdSe/CdS CdSe/CdS/ZnS	4.0 5.5 6.8	Same OD with standard	16% 38% 75%	Rhodamine 6G (95%)	Bare CdS: 2ML ZnS: 2ML	[168]
CdSe CdSe/SiO_2_		OD: 0.01 Excitation: 350 nm (B) 450 nm (G) 500 (R)	22% (bare 523 & 581 nm) 82% (542 nm)	9,10-diphenyl-anthracene in cyclohexane (90% @ 350nm); Fluorescein in 0.1 M NaOH (95% @450 nm); Rhodamin 6G in methanol(95% @ 500 nm)	Shell thickness: ~6nm	[72]

OD: optical density; ML: monolayer; 1 CdS ML [168]: 0.35 nm; 1 ZnS ML [168]: 0.31 nm

**Figure 13:**
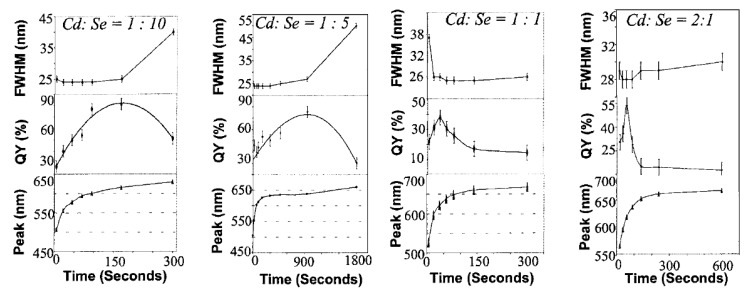
Change of quantum yield with reaction time and growth of particles, and stoichiometric ratio of anion and cation precursors (reprinted with permission from [164]. Copyright 2002 American Chemical Society).

First, we start our discussion with QY data from CdSe Qdots. The QY of CdSe/ZnS core/shell Qdots was measured using rhodamine (RD) 560 in ethanol at excitation 560 nm (emission 480–850 nm) [103]. Integrated emission intensities of RD 590 or RD 640 and Qdots with same OD at excitation wavelength were used to determine QYs of Qdots [104]. QY of CdSe/CdS Qdots was found to be as high as 84% [70]. *Peng et al.* determined QY by comparing integrated intensities of Qdot PL to two standards: RD 6G and RD 640 in methanol at optical densities of all solutions less than 0.3 at the excitation wavelengths [70]. For Blue emitting ZnSe, *Hines et al.* [160] used stilbene 420 in methanol found as high as 50% QY. *Norris et al.* [163], on the other hand, found ~22% QY in room temperature from ZnSe:Mn Qdots. *Peng et al.* [164] described QY measurement process where the optical density at the excitation wavelength was kept same value with Qdots. They also kept ODs at the first exciton absorption peak of Qdots and peak absorption peaks of dyes at below 0.1. Figure 13 is adapted from *Dr. X. Peng*’s highly cited JACS paper [164], where it has been shown that the composition of Qdots and stoichiometric ratio of precursors are important parameters to improve the QY values of Qdots. *Meijerink’s* group [166] from the *Netherlands* reported the same. *Dijken et al.* studied the QY of ZnO Qdots [169]. QYs of the visible emission from ZnO nanoparticles prepared by colloidal solution were found to decrease from 20% to 12% as the particle radius increased from 0.7 to 1 nm [169]. 

The PL-QYs of ZnSe Qdots can be increased by coating with a wider band-gap semiconductor, such as ZnS which has an ~5% lattice mismatch with ZnSe [170]. After passivation with 1.8 monolayers of ZnS shell, the QYs of ZnSe/ZnS core/shell Qdots increased 450% to a value of ~32%. We recently reported QYs of ~5% and 13% for 3–4 nm ZnO Qdots [102] and ZnO/MgO core/shell structure [159], and the QY decreased with aging time in air due to a reduced concentration of radiative traps [159]. *Bol* and *Meijerink* [171] compared the QY for Qdots coated with poly(vinybutryral) (PVB), poly(vinylalcohol) (PVA), methacrylic acid (MA), and sodium polyphosphate (PP), and the QYs of 0.3% to 1% for unpassivated Qdots was increased to ~4% for ZnS:Mn capped with PP. The Mn^2+^ emission at 580nm from ZnS:Mn/ZnS core/shell Qdots was found to be seven times more intense than from unpassivated ZnS:Mn. The enhanced intensity was believed to result from suppression of nonradiative transitions by the undoped ZnS-shell. Qdots of ZnS:Mn coated by a SiO­_2_ shell showed an enhanced PL intensity as compared to bare Qdots [172]. We recently reported [72] the QY of bare CdSe Qdots to be between 9–21% whereas for silica coated Qdot exhibited QY more than 80%. 

##### 3.2.2.2. Change of Quantum Yield under Ultraviolet Irradiation

**Figure 14:**
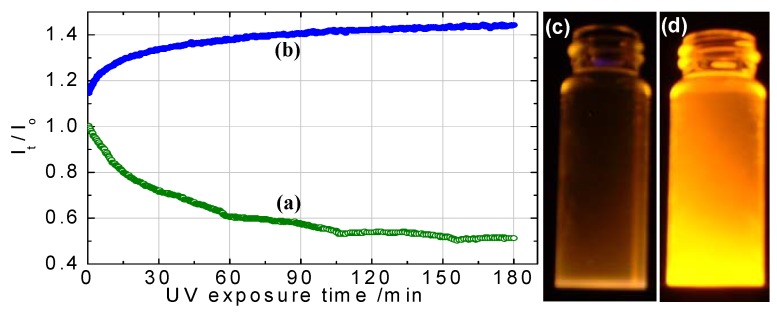
Variation of PL intensity in room temperature air from (a) n-dodecanethiol and (b) ZnS-passivated CdS:Mn Qdots *vs.* time of exposure to 400 nm UV light. The monitored wavelengths are 580 nm and 585 nm for n-dodecanthiol and ZnS passivated CdS:Mn Qdots, respectively. Comparison of relative brightness from (c) n-dodecanthiol passivation and (d) ZnS-passivated CdS:Mn Qdots under 366 nm UV-illumination [180] (reproduced by permission of The Electrochemical Society).

A significant increase in the luminescence QY of ZnS Qdots was observed due to UV irradiation. *Becker* and *Bard* attributed this phenomenon to irradiation-induced oxygen absorption that blocked nonradiative recombination at surface states [173]. *Henglein et al.* proposed [174] that photoanodic dissolution of the ZnS Qdots was induced by irradiation in the presence of oxygen, which led to the improved efficiencies. *Dunstan et al.* explained the increased efficiencies in terms of a photocorrosion process that created new recombination centers [175]. In our group, *Yang, et al.* [97] used XPS data to show that 400 nm irradiation in air converted ZnS shells to ZnSO_4_ which increased the QY of CdS:Mn/ZnS core/shell structures. UV irradiation in argon did not result in the formation of ZnSO_4_ nor did it change the QY. UV irradiation of organically passivated ZnS Qdots can either increase or decrease the QY. *Bhargava et al.* [37] observed an increase upon UV irradiation and speculated that increased cross-linking and polymerization of the passivating organic molecules was the mechanism. Recently, *Bol* and *Meijrink* [176] postulated that enhanced emission from organically passivated surfaces resulted from either UV curing of samples coated with PVB, PVA, or MA, or from a photochemical reaction at Qdot surfaces coated with PP or on unpassivated samples. After UV curing, QYs of ~10% were obtained. However, prolonged UV irradiation (hours to days) in the presence of water and oxygen led to a decreased QY. As in the case of *Yang, et al.* [97], photochemical reactions could produce ZnSO_4_ or Zn(OH)_2_, which presumably served as a passivating layer around Qdots (Figure 14), but too thick a layer can lead to lower QYs. In the case of ZnS:Mn/ZnS core/shell structures, luminescence intensity was not changed significantly as a result of UV irradiation [177]. For ZnS:Mn/SiO_2_ core/shell nanoparticles, UV irradiation increased the PL intensity [172]. The luminescent intensity from ZnS:Mn^2+^ colloid solutions decreased after the colloids were kept in room temperature air [178]. This was presumably due to the deterioration of surface structure, which led to an increase of nonradiative relaxation paths. *Eychmüller et al.* reported a PL peak at ~390 nm from thiol passivated, water soluble, sulfur alloyed ZnSeS colloidal Qdots (~ 2–3nm in size) [179]. UV irradiation after synthesis improved their QY to 25–30% for the band-gap UV emission. Irradiation resulted in incorporation of sulfur into the ZnSeS Qdots. 

#### 3.2.3. Non-radiative Process in Quantum Dots

Absorption of energy by a luminescent material may not result in emission of light. Electrons and holes in excited states may return to lower energy and ground states by radiative and/or nonradiative relaxations. Deep level traps have a tendency to undergo nonradiative recombination by emitting phonons. Experimental data show that the time required for nonradiative recombination is short (e.g., tens of picoseconds [119]). Non-radiative relaxation may be categorized as internal conversion, external conversion or *Auger* recombination [181]. Nonradiative recombination though crystalline and/or molecular vibrations is a common phenomenon in internal conversion. The difference between the energy absorbed by Qdots, hν*,* and the band-gap, E_g_, is generally converted into heat by electron-phonon scattering processes. Even for a radiative process in an indirect semiconductor, a phonon is generated due to the change of k-value. Furthermore, strain in a lattice can create a local potential well that can also trap electrons and holes and result in a nonradiative transition. Nonradiative recombination also occurs at surface states. As reported above, 15–30% of atoms in a Qdot are at the surface and represent defects due to unsaturated dangling bonds. These defects are dominant channels for nonradiative decay of carriers. The electronic surface states are filled below the *Fermi* level with electrons from the core of the Qdots. Accumulation of charge at the surface creates an electric field or a depletion region that leads to bending of the valence and conduction band-edges. Electron and hole carriers generated in this region are swept in opposite direction by the electric field, prohibiting radiative recombination. This leads to the concept of a ‘dead layer’ [125]. Capping of these defects with organic ligands or inorganic shells lead to an improvement in luminescent efficiency as discussed below and in section 2.2.1. 

Strong carrier-to-carrier interaction can lead to an *Auger* nonradiative process (rather than releasing the energy of recombination as a photon or phonon, the excess energy is transferred to another electron). The *Auger* electron loses its surplus energy by creation of phonons. The *Auger* recombination process involves two electrons and a hole in the conduction and valence bands, respectively. (Sometimes *Auger* process involves two holes and one electron). *Auger* recombination can also create a hole deep in the valence band or can be observed for electrons and holes on localized activator levels [181]. In an *Auger* transition, the momentum and energy must be conserved. Therefore, indirect semiconductors show much higher *Auger* recombination rates as compared to direct band-gap materials. This is due to the fact that a momentum change is necessary for *Auger* recombination, and is also required for the transition in indirect band-gap semiconductors. *Auger* process differs greatly between nano-systems and bulk systems of same composition as the efficiency of *Auger* process depends on *Coulomb* electron-electron interaction. In atomic or nano-systems, electron-electron coupling is stronger than the electron-photon coupling. Therefore, the rate of *Auger* transition is higher compared to radiative transition [182]. 

## 4. Synthesis Processes

Several routes have been used to synthesize Qdots. Generally, the techniques for synthesis of Qdots are categorized either as a top-down or bottom-up approach. Below, we discuss both approaches in brief. 

### 4.1. Top-Down Synthesis Processes

In the top-down approaches, a bulk semiconductor is thinned to form the Qdots. Electron beam lithography, reactive-ion etching and/or wet chemical etching are commonly used to achieve Qdots of diameter ~30 nm. Controlled shapes and sizes with the desired packing geometries are achievable for systematic experiments on quantum confinement effect. Alternatively, focused ion or laser beams have also been used to fabricate arrays of zero-dimension dots. Major drawbacks with these processes include incorporation of impurities into the Qdots and structural imperfections by patterning. Etching, known for more than 20 years, plays a very important role in these nanofabrication processes. In dry etching, a reactive gas species is inserted into an etching chamber and a radio frequency voltage is applied to create a plasma which breaks down the gas molecules to more reactive fragments. These high kinetic energy species strike the surface and form a volatile reaction product to etch a patterned sample. When the energetic species are ions, this etching process is called reactive ion etching (RIE). With a masking pattern, selective etching of the substrate is achieved. Fabrication of GaAs/AlGaAs quantum structures as small as 40 nm has been reported using RIE with a mixture of boron trichloride and argon [183]. This RIE process has been used to produce close-packed arrays for testing of lasing in Qdot semiconductors. Close packed arrays of ZnTe Qdots with interdot distance of 180 nm to 360 nm were produced by RIE using CH_4_ and H_2_ [184]. 

Focused ion beam (FIB) techniques also offer the possibility of fabricating Qdots with extremely high lateral precision. Highly focused beams from a molten metal source (e.g., Ga, Au/Si, Au/Si/Be, or Pd/As/B) may be used directly to sputter the surface of the semiconductor substrate. The shape, size and inter-particle distance of the Qdots depend on the size of the ion beam but a minimum beam diameter of 8–20 nm has been reported for both lab and commercial systems, allowing etching of Qdots to dimensions of <100 nm [185]. The FIB technique can also be used to selectively deposit material from a precursor gas with a resolution of ~100 nm. Scanning ion beam images (analogous to scanning electron microscope images) can be developed by ion beam nanofabrication at the desired, predetermined locations with high resolution [185]. However this is a slow, low throughput process employing expensive equipment that leaves residual surface damage. Another method to achieve patterns with Qdots dimensions is the use of electron beam lithography followed by etching or lift-off processes. This approach offers a high degree of flexibility in the design of nanostructured systems. Any shape of Qdots, wires, or rings with precise separation and periodicity may be realized with this technique. This method was successfully employed for the synthesis of III-V and II-VI Qdots with particle sizes as small as 30 nm. 

### 4.2. Bottom-up Approach

A number of different self-assembly techniques have been used to synthesize the Qdots, and they may be broadly subdivided into wet-chemical and vapor-phase methods. Microemulsion, sol-gel, competitive reaction chemistry, hot-solution decomposition, and electrochemistry are generally placed in the category of (1) wet-chemical methods. Self-assembly of nanostructures in material grown by molecular beam epitaxy (MBE), sputtering, liquid metal ion sources, or aggregation of gaseous monomers are generally categorized under (2) vapor-phase methods.

#### 4.2.1. Wet-Chemical Methods

Wet-chemical methods mainly follow the conventional precipitation methods with careful control of parameters for a single solution or mixture of solutions. The precipitation process invariably involves both nucleation and limited growth of nanoparticles. Nucleation may be categorized as homogeneous, heterogeneous or secondary nucleation [186]. Homogeneous nucleation occurs when solute atoms or molecules combine and reach a critical size without the assistance of a pre-existing solid interface. By varying factors, such as temperature, electrostatic double layer thickness, stabilizers or micelle formation, concentrations of precursors, ratios of anionic to cationic species and solvent, Qdots of the desired size, shape and composition can be achieved. Some of the common synthesis processes are briefly discussed below.

##### 4.2.1.1. Sol-Gel Process

Sol-gel techniques have been used for many years to synthesize nanoparticles including Qdots [61,102,133,159]. In a typical technique, a sol (nanoparticles dispersed in a solvent by *Brownian* motion) is prepared using a metal precursor (generally alkoxides, acetates or nitrates) in an acidic or basic medium. The three main steps in this process are hydrolysis, condensation (sol formation) and growth (gel formation). In brief, the metal precursor hydrolyzes in the medium and condenses to form a sol, followed by polymerization to form a network (gel). This method has been used to synthesize II-VI & IV-VI Qdots, such as CdS [12], ZnO [61,102,133,159], PbS [187]. As an example, ZnO Qdots have been prepared by mixing solutions of Zn-acetate in alcohol and sodium hydroxide, followed by control aging in air [61]. The process is simple, cost-effective and suitable for scale-up. The main disadvantages of the sol-gel process include a broad size distribution and a high concentration of defects [187]. Therefore, this synthesis technique is used sparingly.

##### 4.2.1.2. Microemulsion Process

Microemulsion processes are popular methods for synthesizing Qdots at room temperature. The processes can be categorized as either normal microemulsions, *i.e.,* oil-in-water, or as reverse microemulsions, *i.e.,* water-in-oil. In some cases, other polar solvents, e.g., alcohol, may be used instead of water. The reverse micelle process is popular for synthesizing Qdots, where two immiscible liquids (polar water and nonpolar long-chain alkane) are mixed and stirred to form emulsion. Nanometer water droplets dispersed in *n*-alkane solutions can be achieved using surfactants, like aerosol OT (AOT), cetyl trimethyl-ammonium bromide (CTAB), sodium dodecyl sulphate (SDS) or triton-X. Since the surfactants are terminated by hydrophilic and hydrophobic groups on opposite ends, numerous tiny droplets called micelles are formed in the continuous oil medium. These micelles are thermodynamically stable and can act as ‘nanoreactors’. Mixing of vigorous stirred micellar solutions leads to a continuous exchange of reactants due to dynamic collisions. Growth of the resultant Qdots is limited by the size of the micelle which is controlled by the molar ratio of water and surfactant (W). The relation between W and the radius (r) of the micelle has been reported as [188] 

(6) $${\left(\frac{r+15}{r}\right)}^{3}-1=\frac{27.5}{W}$$

The reverse micelle technique has been used to prepare II-VI core and core/shell Qdots, such as CdS [63], CdS:Mn/ZnS [76,97,189,190], ZnS/CdSe [13], CdSe/ZnSe [188], ZnSe [115] and IV-VI Qdots [108]. Some advantages of this process are easy control of the Qdot size by changing the molar ratio of water to surfactant, a narrow distribution of size as compared to the sol gel process, and ease of dispersion of the Qdots. Some disadvantages include low yield and incorporation of impurities and defects.

##### 4.2.1.3. Hot-Solution Decomposition Process

High temperature (~300 °C) pyrolysis of organometallic compound is a well-established route for the production of Qdots and was first discussed in detail in 1993 by *Bawendi* and co-workers [14]. Precursors, such as alkyl [14], acetate [191], carbonate [191] and oxides [164,191] of Group II elements, are mixed with Group VI phosphene or bis(trimethyl-silyl) precursors. A typical procedure involves first degassing and drying of trioctyl-phosphine oxide (TOPO, a coordinating solvent) at 200–350 °C under vacuum (1 Torr or 7.5 × 10^-6^ Pa) in a three-neck round flask in a dry box. A mixture of Cd-precursor and tri-*n*-octyl-phosphine (TOP) selenide is prepared in a dry box and injected with vigorous stirring into the flask at a temperature of ~300 °C. The simultaneous injection of precursors into the flask along with TOPO results in homogeneous nucleation to form Qdots, with the subsequent growth of Qdots through ‘*Ostwald ripening*’ being relatively slow. In *Ostwald ripening*, the higher free energy of smaller Qdots makes them lose mass to large size Qdots, eventually disappearing. The net result is a slow increase of the size of Qdots at the reaction temperature of ~230*–*250 °C (depending on precursor, coordinating agents and solvents). The coordinating TOPO solvent stabilizes the Qdot dispersion, improves the passivation of the surface, and provides an adsorption barrier to slow the growth of the Qdots. The final size of the Qdots is mainly controlled by the reaction time and temperature. Aliquots may be removed from the flask at regular intervals during the first few hours and the optical absorption edge used to achieve a desired particle size. This method has been extensively used to synthesize II-VI [56,111,160,192,193,194,195,196], IV-VI [197] and III-V Qdots [198]. The size, shape, and control of the overall reaction depends not only on process parameters and precursors, solvents and coordinating agents, but also on the purity of the coordinating solvent, such as TOPO. It has been reported that technical grade TOPO (90% pure) were better for synthesizing uniform Qdots than the pure TOPO [162,191]. 

An advantage of this synthesis route is that it provides sufficient thermal energy to anneal defects and results in monodispersed Qdots (typically standard deviation about the average size of 5%). Since growth of the particles in this process is relatively slow and can be controlled by modulating the temperature, a series of Qdot sizes can be prepared from the same precursor bath. Using this process large quantities of Qdots [199] and alloying process [52] have been demonstrated. Some of the disadvantages of this method include higher costs due to the use of high temperature, toxicity of some of the organometallic precursors, and generally poor dispersions in water. Table 3 shows a chronological summary of this synthesis technique with different growth parameters and precursors to produce different Qdots. 

**Table 3:** Synthesis of different sized Qdots using hot-solution decomposition reaction.

Year	Qdots	Precursor	Process parameters	Particle Size (nm)	Ref
1990	GaAs	GaCl_3_, (TMS)_3_As in Quinoline	240 °C for 3days; flame anneal at 450 °C	2.4	[200]
1990	ZnS, ZnSe, CdS, CdSe, CdTe, HgTe	M(ER)_2_; R: n-butyl phenyl; E: S, Se, Te; M: Cd, Zn, Hg and/or phosphine complexes; Co.Sol.: DEPE	DEPE and M(ER)_2_ reacted, (Temp. range: 250–300 °C)	2.5–5 nm	[201]
1993-1998	CdS, CdSe, CdTe	Me_2_Cd, silylchalconides, Phosphine chalconides; Co.sol: TOPO & TOP/TBP	300–350 °C at 1 atm at Ar (TOPO degassing); 230–260 °C (growth temp.)	1.2–11.5	[14,101,202]
1994	GaAs	GaCl_3_/ GaI_3_, diglyme, As, toluene, Na-K alloy	As, Na-K alloy mixture refluxed to 100 °C in Ar for 2 days; GaCl_3_/GaI_3_ diglyme mixture added, heated from 0 °C to RT to 111 °C. for 2 days	6–10	[203]
1995	InP, GaP, GaInP_2_	Mixture of chloro-indium/gallium oxalate (GaCl_3_ for GaP) and (TMS)_3_P in CH_3_CN ; Co.sol: TOPO & TOP	270–360 °C at airless condition for 3 days; Qdots dispersed in methanol	2.6–4.6 (InP), 3 (GaP), 6.5 (GaInP_2_)	[204]
1996	InP, InAs	InCl_3_, TOPO, (TMS)_3_P/(TMS)_3_As	InCl_3_ & TOPO heat at 100 °C for 12 h, (TMS)_3_P added, after 3hr heated to 265 °C for 6 days	2–6	[205,206]
1996	CdSe/ZnS	Me_2_Cd,Me_2_ Zn, Se, (TMS)2S, Co.sol: TOPO, TOP	Single step synthesis Core: 350 °C at 1 atm at Ar, growth: 310 °C Shell: 300 °C	2.7–4	[103]
1997	CdSe/ZnS	Me_2_Cd, Me_2_ Zn, Se, (TMS)2S, Co.sol: TOPO, TOP	Two step synthesis (airless) Core growth: 290–300 °C Shell growth: 140 °C for 2.3 nm & 220°C for 5.5 nm	2.3–5.5	[104]
1997	CdSe/CdS	Me_2_Cd, Se, (TMS)2S, Co.sol: TOPO, TBP	Two step process: Core: 300 °C; Shell: 100 °C	2.5–4	[70]
1998	ZnSe	Me_2_Zn, Se, HDA, TOP	HDA dried & degassed at 150 °C for hrs in vacuum and heated to 310 °C at 1 atm in Ar; Core growth with Zn & Se precursor at 270 °C.	4.3–6 nm	[160]
1996–1999	InAs/InP InAs/CdSe	(TMS)_3_As, Indium (III) chloride, TOP (TMS)3P, Me_2_Cd; TBP-Se	Two-step Process (airless) Core growth: 260 °C; Shell: dropwise addition; 260 °C	2.5–6 nm (InAs); 1.7 (core/shell)	Core [202,206] Core/shell [207]
2000	CdSe	Me_2_Cd, Se, TBP, TOPO, HPA	TOPO (+HPA 1.5–3 wt%) degassed at 360 °C (or 310 °C, 280 °C); Core growth: 300 °C (or 280 °C or 250 °C)	~6nm	[208,209]
2001	ZnSe:Mn	Me_2_Mn, Et_2_Zn, TOP, Se, HDA	Dimethyl Mn, TOP, Se, Diethyl Zn mixture added to HDA at 310 °C in N­_2_. Growth: 240–300 °C	2.7–6.3	[163]
2001- 2003	CdSe/ZnS	Me_2_Cd, Se, TOP, TOPO, HDA, (TMS)2S, Me_2_Zn	Two Step: Core: reaction & growth: 270–310 °C; Shell: slow addition of Zn & S precursor at 180–220 °C	4.5–5 nm	[162,210]
2001	CdSe	Scheme 1: Cd(Ac)_2_,, SA/ TOPO; 2: Cd(Ac)_2_, SA; 3: CdCO_3_, SA/TOPO; 4: CdCO_3_, LA/TOPO; 5: CdO, SA/TOPO; 6: Cd(Ac)_2_, tech TOPO; 7: CdO, TDPA/TOPO	Solvent & Cd-precursor heated to 250–360 °C at Ar; TOP-Se or TBP-Se injected; Growth temp: 200–320 °C (if DDA involve, temp: ~220 °C	2–25nm	[191]
2001	CdS, CdSe, CdTe	CdO, TOPO, HPA/TDPA, S, Se, Te & TOP	One pot: CdO, HPA/TDPA heated 300 °C; Core with chalconide precursor: reaction: 270 °C, & growth 250 °C	2–8 nm	[211]
2002	CdSe	CdO, Se, TOPO, TBP, HDA, ODA, SA	CdO & SA, heated to 150 °C in Ar; after CdO dissolution, cool to RT; TOPO & HAD added & heated to 320 °C in Ar; TBP-Se added, Growth 290 °C		[164]
2003	PbS	PbO, OA, (TMS)_2_S, TOP	PbO dissolved in oleic acid at 150 °C in Ar; (TMS)_2_S & TOP injected	5nm	[197]
2003	CdSeS	CdO, OA, TOA, Se, S, TOP	CdO+ OA+TOA heated at 300 °C in N_2_, TOP-S, TOP-Se injected	~5nm	[26]
2005	PbSe	Pb-acetate trihydrate, OA, Se, TOP	Single Step: Pb acetate + Co.sol degassed at 100–120 °C at 300–500 mTorr for 2h; reaction and growth: 140 °C	5 nm	[212]
2006	CdSe	CdO, OA, TOA, C_8_SH or C_18_SH,	CdO + OA + TOA heated at 300 °C; TOA + C_8_SH or C_18_SH injected	3, 4, 6 nm	[213]

Ac: acetate; Co.sol: coordinating solvent; DDA: dodecylamine; DEPE: 1,2-bis(diethyl-phosphino)-ethane; DMPA: 2,2 –dimethoxy-2-phenylacetophenone; EGDMA: ethylene glycol domethacrylate; Et: ethyl; HDA: hexadecylamine; HPA: hexyl-phosphonic acid; LA: lauric acid; Me: methyl; MMA: methylmethacrylate; MPA: marcaptopropionic acid; OA: oleic acid; ODA: octadecylamine; ODE: 1-octadecene; SA: stearic acid; TBP: tri-n-butyl phosphine; TDPA: tetradecylphosphonic acid; TMS: trimethyl-silyl; TOA: trioctyl amine; TOP: tri-n-octyl-phosphine; TOPO: tri-n-octyl-phophine oxide (tech TOPO: Technical grade TOPO) 1 Torr = 7.5 × 10^-6^ Pa

##### 4.2.1.4. Other Synthesis Processes

Sonic waves or microwaves [214,215] have been passed through a mixture of precursors in water to grow Qdots. These waves provide energy to dissociate the precursor and water molecules which results in the growth of Qdots. Ultrasound waves have been reportedly used to synthesize Qdots in the size range of 1–5 nm by formation, growth and implosive collapse of bubbles in a liquid [214]. Such acoustic cavitation generates a localized hotspot through adiabatic compression within the gas inside the collapsing bubble, enabling the reactions that form Qdots. In one approach, acetate precursors of metal ions were dispersed in a solution and seleno-urea was added and sonicated for an hour under an argon atmosphere [214]. The temperature of the solution rose to 80 °C during the time required to produce Qdots.

Hydrothermal synthesis methods [216,217] or similar synthesis process [218] have been used to produce Qdots. These are crystallization of inorganic salts from aqueous solution, controlled by pressure and temperature. The solubility of inorganic compounds typically decreases as the temperature and/or pressure is lowered, leading to crystalline precipitates. By changing pressure, temperature, reaction and aging time and reactants, different shapes and sizes of the Qdots can be achieved. Several other wet-chemical processes for synthesizing Qdots [28,219,220] were also reported in literatures. Passing H_2_S gas through precursors has also been used to prepare sulfide-based Qdots [221,222].

#### 4.2.2. Vapor-Phase Methods

Initially, *in situ* self-assembled nanostructured materials were produced by hetero-epitaxial growth of highly strained materials. These vapor-phase methods for producing Qdots begin with processes in which layers are grown in an atom-by-atom process. Consequently, self-assembly of Qdots occurs on a substrate without any patterning [223,224,225,226,227]. In general, the layered materials grow as a uniform, often epitaxial layer (*Frank-van der Merwe* mode*–*FvdM) [228], initially as a smooth layer sometimes followed by nucleation and growth of small islands (*Volmer-Weber* mode*–*VW) [229], or as small (Qdots) islands directly on the substrate (*Stranski-Krastonow* mode*–*SK) [230]. Depending on the interfacial/surface energies and lattice mismatch (*i.e.,* lattice strain), one of these growth modes is observed. For example, Qdots may be formed by SK growth with an overlayer material that has a good lattice match with the substrate, but the substrate surface energy (σ_1_) is less than the sum of the interfacial energy between the substrate and overlayer (γ_12_) and the overlayer surface energy (σ_2_), *i.e.,* when *σ_1 _< σ_2_+γ_12_* [231]. In other cases, formation of Qdots was due to relaxation of strain required to maintain epitaxy. In the case of substrates with an overlayer with a large lattice mismatch and appropriately small surface and interface energies, initial growth of the overlayer occurs through by a layer-by-layer FvdM growth. However, when the film is sufficiently thick (a few monolayers) to induce a large strain energy, the system lowers its total free energy by breaking the film into isolated islands or Qdots (*i.e.,* the VW mode). *Kim et al.* [232] synthesized ZnSe/ZnS Qdots with the SK growth mode using a metal-organic chemical vapor deposition (CVD) technique in an atomic layer epitaxy (ALE) mode. The mean dot height was 1*–*1.9 nm. An apparent temperature dependent, anomalous behavior of confined carriers in the ZnSe Qdots was observed and attributed to thermalized carrier hopping between Qdots. The carrier hopping resulted in a substantial decrease of the PL peak energy and line width when the sample was cooled from room temperature to 140 K. 

Molecular beam epitaxy (MBE) has been used to deposit the overlayers and grow elemental, compound or alloy semiconductor nanostructured materials on a heated substrate under ultra high vacuum (~ 10^-10^ Torr or 7.5 × 10^-16^ Pa) conditions [233,234]. The basic principle of the MBE process is evaporation from an apertured source (*Knudsen* effusion cell) to form a beam of atoms or molecules. The beams in the MBE process can be formed from solids (e.g., elemental Ga and As are used to produce GaAs Qdots) or a combination of solid plus gases (e.g., AsH_3_, PH_3_, or metal-organics such as tri-methyl gallium or tri-ethyl gallium). The metal-organic sources may leave high concentrations of carbon in the Qdots. MBE has been mainly used to self-assemble of Qdots from III-V semiconductors [223] and II-VI semiconductors [224,235] using the large lattice mismatch e.g., InAs on GaAs has a 7% mismatch and leads to SK growth, as discussed above. 

Layer growth by physical vapor deposition (PVD) results from condensation of a solid from vapors produced by thermal evaporation or by sputtering [186]. Different techniques have been used to cause evaporation, such as electron beam heating, resistive or *Joule* heating, arc-discharge and pulsed laser ablation. In any case, the factors discussed above (strain and surface energies) control the formation of Qdots from the deposited thin films. CVD is another method to form thin films from which Qdots can be self-assembled. In CVD, precursors are introduced in a chamber at a particular pressure and temperature and they diffuse to the heated substrate, react to form a film, followed by gas-phase byproducts desorbing from the substrate and being removed from the chamber. InGaAs and AlInAs Qdots have been synthesized using either surface energy or strained-induced SK growth processes [236]. Although, self-assembling of Qdots using vapor-phase methods is effective in producing Qdots arrays without template, fluctuation in size of Qdots often results in inhomogeneous optoelectronic properties.

## 5. Application 

### 5.1. Quantum Dots for Electroluminescence Device Fabrication

Qdots-based light emitting diodes (QLEDs) have attracted intense research and commercialization efforts over the last decade [22]. Infact, QLEDs have several advantages compared to organic LEDs (OLEDs). These are as follows: (i) FWHM of the emission peak from Qdots is only 20–30 nm, compared with >50 nm for their organic counterpart, which is necessary for a high quality image; (ii) Inorganic materials usually show better thermal stability than organic materials. Under operating at high brightness as well and/or high current, *Joule* heat is one of the predominant problems for device degradation. With better thermal stability, inorganic materials based devices are expected to exihibit longer lifetimes. (iii) The display color of OLEDs generally changes with time due to the different lifetime of the red, green and blue pixels [237]. However, one can obtain all of three premium colors from Qdots with the same composition changing the particle size (due to the quantum confinement effect, as discussed above). The same chemical composition should exhibit similar degradation with time. (iv) The QLED device can produce IR emission while the organic molecules in OLEDs usually exhibit wavelengths shorter than 1 μm. (v) The spin statistics are not restrictive for Qdots, *i.e.,* external quantum efficiency (EQE) of 100% can be achieved (in principle). The EQE of QLEDs can be expressed [238] as: $\eta_{Ext}=\eta_{r}\cdot \eta_{INT}\cdot \eta \cdot \eta_{OUT}$, where, η_r_ is the probability of holes and electron forming exciton, η*_INT_* is the internal PL-QY and η & η_OUT_ are the probability of radiative decay and out-coupling efficiency, respectively. The value of η_r_ for fluorescent organics theoretically is limited to 25% as the ratio of singlet to triplet states is 1:3 and only singlet state recombinations result in luminescence. However, for phosphorescent organics it is found > 25% due to spin-orbit coupling [239]. Note that phosphorescent organics lead to rapid degradation of the host material. With respect to high η_Ext_, the value of η_OUT_ for planar devices is typically found to be ~20% [240]. The η_OUT _efficiency can be enhanced by incorporating a microcavity structure [241]. For QLEDs, the value of η*_INT_* (QY) can approach 100% and for a device with the appropriate electron and hole energies, the value of η_r_ can also be ~100%. It has been observed that a QLED can emit light under both forward and reverse bias [220]. Reason for this behavior is uncertain and many explanation are plausible, such as, different rates of electron and hole injections, different carrier mobility in the electron and hole transport layers, energy level offsets of the different layers, the emitting layer composition, surface and, uniformity and thickness of the layers. The valence (HOMO) and conduction (LUMO) band energies of some of the polymers that have been used in inorganic/organic QLEDs and solar cell are tabulated in Table 4. Table 5 shows the valence band and conduction band-energies reported in literature for different sized and structured Qdots, and Table 6 shows the work functions of some of the commonly used materials in QLED solar cell and OLEDs.

**Table 4:** Valence band (HOMO) and conduction band (LUMO) energies for some of the commonly used organics for QLEDs, solar cells and OLEDs.

Organics	Conduction Band (eV)	Valence Band (eV)	Reference
Alq3	3.1	5.8	[242,243]
CBP	2.9	6.0	[244]
PBD	2.6	6.1	[245]
PCBM	4.0	6.5	[246]
PPV	2.5	5.1	[247,248]
PVK	2.2	5.3	[245]
TAZ	3.0	6.5	[249]
TFB	2.2	5.4	[250]
TPBI	2.7	6.2	[244,251]
TPD	2.1	5.4	[242]
Poly TPD	2.3/2.5	5.2/5.4	[244,252]

Alq3: tris-(8-hydroxyquinoline) aluminum; CBP: 4,4’,N,N’-diphenylcarbazole; t-Bu-PBD: 2-(4-biphenylyl)-5-(4-tert-butylphenyl)-1,3,4 oxadiazole; PCBM: [6,6]-phenyl C61 butyric acid methyl ester; PPV: poly(phenylene vinylene); PVK: poly(vinyl-carbazole); TAZ: 3-(4-Biphenylyl)- 4-phenyl-5-tert-butylphenyl-1, 2, 4-triazole; TFB: Poly[(9,9-dioctylfluorenyl-2,70diyl)-co-(4-4’-(N-(4-sec-butylphenyl)) diphenylamine)]; TPBI: 1,3,5-tris(N-phenylbenzimidazole-2-yl)-benzene; TPD: N, N’-diphenyl-N, N’-bis(3-methylphenyl)-(1, 1’-biphenyl)-4, 4’-diamine;

**Table 5:** Valence band and conduction band energies for different sized and structured CdSe Qdots.

Qdots	Conduction Band (eV)	Valence Band (eV)	Particle Size (nm)	Emission	Ref.
CdSe	4.4	6.5	5		[248]
CdSe/CdS	4.4	6.5	4.6		[251]
CdSe/CdS	4.7	6.8	4		[253]
CdSe/CdS/ZnS	4.8	6.8	6.8	600	[250]
CdSe/ZnS	4.4	6.5			[247]
CdSe/ZnS	4.3	6.5		550 nm	[242]
CdSe/ZnS	4.8	6.5			[245]
CdSe/ZnS	4.6 (CdSe)	6.8 (CdSe)	5.8		[249]
CdSe/ZnS	4.7	6.7			[254]
CdSe/ZnS/CdS	3.9	6.0	3–8.3	G, Y, O, R	[252]

**Table 6:** Work function of some of the commonly used materials as anode or cathode in QLED, solar cell and OLEDs.

Materials	Work function (eV)
Al	4.1
Ag	4.6
Ca	2.9
ITO	4.7
LiF/Al	2.8
Mg	3.7
PEDOT:PSS	5

ITO: indium tin oxide; PEDOT:PSS: poly(3,4-ethylene-dioxy-thiophene) poly(styrene-sulfonate).

**Figure 15:**
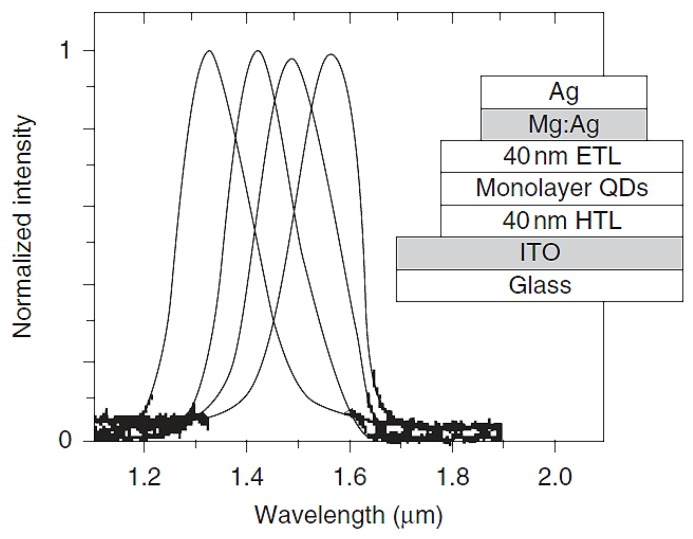
PbSe Qdots based Qdot-LED device structure and NIR-EL spectra using different sized Qdots (ETL: electron transport layer; QDs: quantum dots, HTL: hole transport layer, ITO: indium tin oxide) [Reproduced with permission from [165] Copyright Wiley-VCH Verlag GmbH & Co. KGaA].

Early Qdots-based EL devices were fabricated from self-assembled Qdots, grown using vapor-phase processes. These self-assembled structures were used to study the device physics [255]. *Nakamura et al.* [235] fabricated an EL device with a structure of In/n-GaAs/n-ZnSe:Cl (1μm)/self-assembled CdS/pZnSe:N (1 μm)/Au. The blue-green emission peak wavelength varied with position in the wafer, possibly due to inhomogeneity of the CdS Qdot dimension. Recently, QLEDs have been developed for display and lighting sources [22,242,250,252,256,257,258,259,260,261] and device structure and reported efficiency are tabulated in Table 7. In 1994, *Colvin et al.* [256] demonstrated a Qdot-polymer based hybrid EL device. They used spin-coated poly(phenylene vinylene) (PPV) polymer as the hole transport layer with indium tin oxide (ITO) and Mg as anode and cathode, respectively. The turn-on voltage was 4.0 V and EQE was 0.001–0.01. PPV was also used by many others for the fabrication of QLED devices [247,248,253,262,263], where the Qdots layers were used as an emitting (EML) as well as a charge transport layer, but the EQE was low (<1%). *Coe et. al* [242] created Qdot monolayer by spin-coating TOPO capped CdSe/ZnS core/shell Qdot along with a N,N’-diphenyl-N,N’ bis(3-methylphenyl)-(1,1’-biphenyl-4,4’-diamine (TPD) layer followed by a 40 nm thermally evaporated layer of tris-(8-hydorxyquinoline) aluminum (Alq3) as electron transport layer (ETL). They reported 2000 cd/m^2^ brightness at 125 mA/cm^2^. *Tessler et al.* [264] reported NIR (1300–1400 nm) emitting QLEDs from InAs/ZnSe core/shell Qdots in a multilayer hybrid device structure: ITO//poly(3,4-ethylene-dioxy-thiophene (PEDOT)//(mixture of InAs/ZnSe Qdots and poly[2-methoxy-5-(2-ethylhexyloxy)-1,4-phenylenevinylene] (MEHPPV) or poly[(9,9-dihexylfluorenyl-2,7-diyl)-co-(1,4-{benzo-[2,1’,3]thiadiazole})] (F6BT))//Mg:Al. Although such devices exhibited EQE values of approximately 0.5%, the turn-on voltage was high (15 V). In 2005, NIR emission from an EL device was achieved using monodispersed 5 nm sized PbSe Qdot [165]. Phase segregation during spin-coating was used to fabricate hexagonal close packed Qdots/organic double heterojunction devices. They also introduced an innovative solvent drying process [212,249] during fabrication of QLED device in order to get phase separation and consequently to achieve two distinct layers of polymer and Qdot-monolayer from the mixture. The organic film served as a hole transport layer (HTL) in the QLED and produced the spectra shown in Figure 15. A broad NIR emission (1200 nm- >1700 nm) [220] with an EQE of 0.02% (maximum light output 150 nW/mm^2^ at 50 mA and 2.5 V) was also achieved from a device with the structure shown in Figure 15 (ITO//poly(3,4-ethylene-dioxy-thiophene) poly(styrene-sulfonate) (PEDOT:PSS)//HgTe (4.6 nm)//Al). 

**Figure 16:**
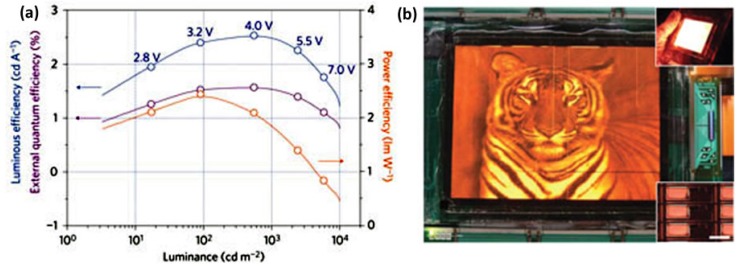
(a) Efficiencies *vs.* luminance data from red emitting QLED devices fabricated using CdSe Qdots; (device exhibited maximum luminance of 12380 cd/m^2^, turn-on voltage of 1.9V and power efficiency of 2.41 lm/W) (b) four inch active matrix QLED display (320 ° 240 pixel array) using a-Si TFT [Reprinted by permission from Macmillan Publishers Ltd. [Nature Photonics] [250], Copyright 2009].

**Figure 17:**
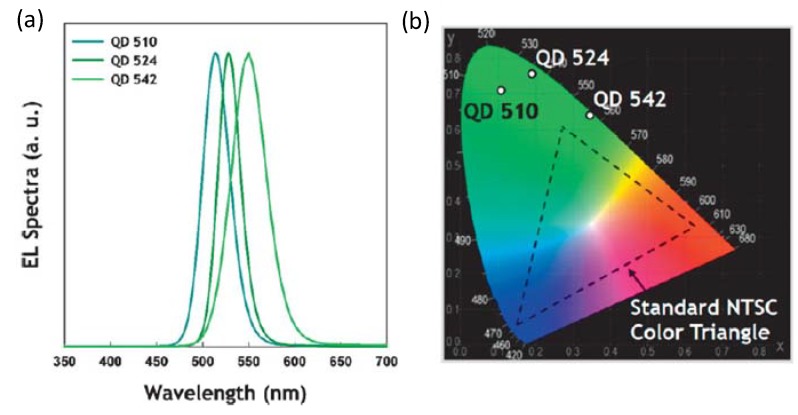
(a) Electroluminescence spectra at 510 nm, 524nm and 542 nm from three different sized CdSe/ZnS Qdots (QD 510: 6.7 nm; QD 524: 7.4 nm; QD 542: 7.8 nm; Maximum brightness obtained from the green emitting device was above 10,000 cd/m^2^. (b) Positions of emitted green colors on the color coordinate (CIE) diagram [Reproduced with permission from [268]. Copyright Wiley-VCH Verlag GmbH & Co. KGaA].

**Figure 18:**
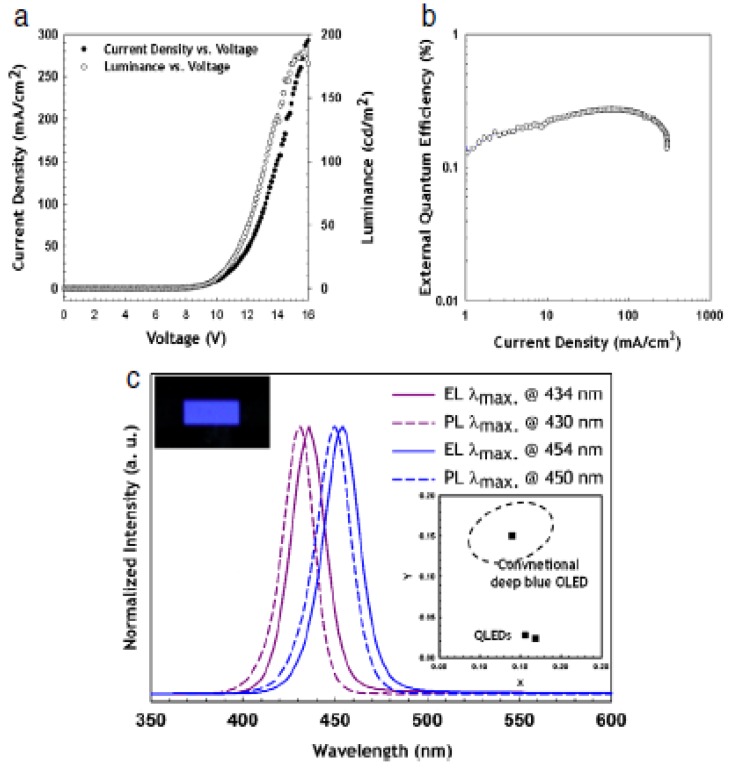
Characteristics of blue emitting QLED using CdZnS/ZnS Qdots: (a) L-I-V characteristics of QLED (b) EQE *vs.* current density and (c) EL and PL spectra from devices. Insets in (c) show a blue emitting electroluminescence device pixel and CIE color coordinate of the QLED [244] (Reproduced with permission from IOP Publishing Ltd).

In another study of QLEDs, *Coe-Sullivan et al.* [249] used thermal evaporation to deposit 30 nm of 3-(4-Bi-phenylyl)-4-phenyl-5-tert-butylphenyl-1,2,4-tru-azole (TAZ) as a hole blocking layer and a 30 nm thick Alq3 layer onto a monolayer of CdSe/ZnS Qdots followed by a Mg:Ag/Ag cathode. Thermal evaporation of the organic layers offsets some of the advantages of QLEDs and has discouraged commercialization of QLEDs. For instance, Alq3 as an ETL and calcium as an electron injection layer was used to obtain an efficient color QLED [252]. Unfortunately, the device was unstable due to degradation of the organic layer and oxidation of the reactive metal cathode [265,266]. In addition, this device fabrication requires a costly batch vacuum deposition process [254]. Furthermore, defects are always present at the organic-inorganic interface which reduces the efficiency. Also, the electron transport rate and intrinsic electron density of an organic thin film is low, leading to an electron density much lower than the hole density and therefore charge imbalanced and again lower efficiencies. Inorganic transport layers were employed to overcome some of these disadvantages. For examples, a NiO layer was used for HTL in an EL device [243,257], because it is chemically and electrically stable and compatible with CdSe Qdots. *Zhao et al.* [251] demonstrated a solution processed TPD based polymer composite HTL where the composite avoided TPD’s tendency to recrystallize during device operation. TPBI (1,3,5-tris(N-phenylbenzimidazole-2-yl)-benzene) ETL was deposited on a monolayer of 4.6 nm CdSe/CdS Qdots using a thermal evaporation method. A monolayer of CdS/ZnS Qdots (QY: 20–30%) exhibited blue EL from a device structure of ITO// 4,4’-N,N”-dicarbazolyl-biphenyl (CBP)//CdS/ZnS Qdots monolayer// 3-(4-biphenylyl)-4-phenyl-5-*tert*-butylphenyl-1,2,4-triazole (TAZ)// Alq3//Mg:Ag//Ag. But the quantum efficiency was only 0.1%. At low current, emission was dominated by the CdS/ZnS Qdots with a FWHM of 30 nm and a peak at 468 nm. At higher currents, EL emission from the organic layers dominated. Using Alq3 as the ETL, *Sun et al.* [252] recently reported red (600 nm), orange (589 nm), yellow (546 nm) and green (517 nm) emitting QLEDs from an organic inorganic hybrid structure using highly luminescent (QY>70%) CdSe/ZnS and CdSe/CdS/ZnS core/shell Qdots. The maximum luminance values for red, orange, yellow and green were 9064, 3200, 4470 and 3700 cd/m^2^, respectively. *Caruge* *et al.* [257] developed a fully inorganic QLED with good long term stability by using a sputtered deposited ZnO:SnO_2_ amorphous layer ETL and alloyed ZnCdSe Qdots instead of core/shell Qdots. Although the QLED showed a maximum luminescence value of ~2000 cd/m^2^ with an EQE of 0.1%, the charge transporting layers were fabricated using vacuum sputter deposition. A blue emitting solution processed QLED device with a maximum brightness 1600 cd/m^2^ and efficiency 0.5 cd/A was reported by *Tan et al.* [267]. *Cho et al.* suggested [250] that the high valance band energy (>6.5 eV) for II-VI Qdots *versus* ITO (4.5–5.1 eV) was the cause of the low efficiency of QLED devices. Using solution-based fabrication steps (except for the Al cathode), they demonstrated a QLED-based display (as shown in Figure 16) having a crosslinked Qdot emitting layer, a HTL and a continuous TiO_2_ thin film ETL. They reported a turn-on voltage of these devices of 1.9 V, lower than the band-gap of the Qdots (2.1 eV). *Bae et al.* [268] recently reported a brightness of more than 10,000 cd/m^2^ from a green emitting QLED (shown in Figure 17) with a turn-on of 3.5V and luminous efficiency of 5.2 cd/A. They also reported a blue emitting QLED using CdZnS alloy Qdots (Figure 18). Finally, we have fabricated hybrid and completely inorganic QLEDs using solution processing (except for the Al cathode) and a unique ZnO nanoparticle as an ETL. We have achieved remarkable luminance and efficacy values for red green and blue emitting devices (yet to be published). Nevertheless, we have tabulated some of the research results in Table 7.

**Table 7:** Literature reported QLED device structures and their efficacy values.

Emitting Wavelength (nm)	V_turn-on _(V)	L_max _(nit)	LE (cd/A)	PE (lm/W)	EQE (%)	Device Structure	Ref.
~610	4	100	--	--	0.001–0.01%	*ITO//CdSe//PPV//Mg & ITO//PPV//CdSe//Mg*	[256]
520, 550, 610	--	--	--	--	0.0005%	*ITO//(mixture PVK – t-Bu-PBD- CdSe)//Al*	[262]
560	4	600			0.2%	*ITO//PPV//CdSe/CdS// MgAg*	[253]
600	3–3.5				0.1%	*ITO//PPV (multilayer) //CdSe//Al*	[247]
620	3.5				0.01–0.2%	*ITO//PPV//(CdSe in block co-polymer)//Al*	[248]
550–650	2.3–3.5				0.1%	*ITO//(mixture of PDDA & CdTe)//Al*	[269]
560	~3.5	2000	1.9	--	0.52%	*ITO//TPD//CdSe/ZnS//Alq_3_//Mg:Ag* *ITO//TPD//CdSe/ZnS//TAZ//Alq_3_//Mg:Ag*	[242]
~580		13			0.005%	*ITO//(mixture of CdSeS & PDBD)//TAZ//Alq_3_//LiF//Al*	[26]
1300–1400	15				0.5%	*ITO//PEDOT//(Mixture of InAs/ZnSe & MEHPPV or F6BT)//Ca:Al*	[264]
540–635	3.5	--	--	--	1.1%	*ITO//TPD//CdSe/ZnS(monolayer)//TAZ//Alq_3_//Mg:Ag//Ag*	[249]
1330–1560	~3				0.001%	*ITO//TPD or αNPD//PbSe//Alq_3_//BCP// Mg:Ag//Ag*	[165]
1000–1500					1.2%*	*ITO//(mixture of PbS & MEHPPV or CNPPV)//Mg//Ag*	[197]
~610	5	500			0.2%	*ITO//PVK//CdSe/ZnS//bu-PBD//Al*	[245]
615	3	7000	2.0	1.0	2%	*ITO//TPD//CdSe//Alq_3_//Mg:Ag//Ag*	[212]
573–619					0.001–0.1%	*Au//pGaN//CdSe/ZnS//nGaN//In*	[254]
1590	~1.2				0.02%	*ITO//PEDOT:PSS//HgTe// Al*	[220]
610	4	1000			1.2%	*ITO//PS-TPD-PFCB//TCTA-BVB//CdSe/CdS//TPBI//Ca//Ag*	[251]
625		3000			0.18%	*ITO//NiO//CdSe/ZnS// Alq3//Ag:Mg//Ag*	[243]
520	2.5				0.5%	*ITO//CBP//CdZnSe/CdZnS//TAZ//Alq3//Mg:Ag//Ag*	[27]
~440 (B) ~545 (G) ~610 (R)	2–3	830 (W)	0.9 (W)	0.57 (W)	0.35% (B) 0.65% (G) 1.6% (R) 0.36% (W)	*ITO//PEDOT:PSS//TPD//Qdots//TAZ//Alq3//Mg/Ag//Ag*	[167]
517 (G) 546 (Y) 589 (O) 600 (R)	4 (G), 5 (Y), 3 (O), 3 (R)	3700 (G), 4470 (Y), 3200 (O), 9064 (R)	1.1–2 (G-R)	<1.1		*ITO//PEDOT-PSS//poly-TPD//CdSe/ZnS or CdSe/CdS/ZnS//Alq_3_//Ca/Al*	[252]
460	2.5	1,600	0.5	0.5	0.06	*ITO//PEDOT:PSS//poly-TPD//CdS/ZnS//Al*	[267]
638	3.8	1950			0.1%	*ITO//NiO//ZnCdSe//ZnO:SnO_2_//Ag*	[257]
~600	1.9	12,380	1.67			*ITO//PEDOT:PSS//TFB//CdSe/CdS/ZnS//TiO_2_//Al*	[250]
434–450	5	150	--	--	0.1–0.3	*ITO//PEDOT:PSS//poly-TPD:CBP//CdZnS/ZnS// TPBI//LiF//Al*	[244]
510, 524, 542	3.5	16,000	6.0	--	1.4	*ITO//PEDOT:PSS//poly-TPD//CdSe/ZnS//TPBI//LiF //Al*	[268]

*Internal quantum efficiency value reported V_turn-on_: turn-on voltage of the device; L_max_: maximum luminance value reported; LE: luminous efficiency; PE: power efficiency; EQE: external quantum efficiency. Alq3: tris-(8-hydroxyquinoline) aluminum; BCP: bathocuproine; CBP: 4,4’-N,N’-dicarbazolyl-biphenyl; CNPPP: poly(2-(6-cyano-6’-methylheptyloxy)- 1,4- phenylene); F6BT: poly[(9,9-dihexylfluorenyl-2,7-diyl)-co-(1,4-{benzo-[2,1’,3]thiadiazole})]; ITO: indium tin oxide; MEHPPV: poly[2-methoxy-5-(2-ethylhexyloxy)-1,4-phenylenevinylene]; αNPD: 4,4-bis[N-(1-naphyl)-N-phenylamino]biphenyl; t-Bu-PBD: 2-(4-biphenylyl)-5-(4-tert-butylphenyl)-1,3,4 oxadiazole; PEDOT: poly~3,4-ethylenedioxythiophene; PFBD: poly(9,9’-dioctylfluorene-co-N-(4-butylphenyl)diphenylamine; PFCB: perfluorocyclobutane; PPV: poly(phenylene vinylene); PS: polystyrene; PSS: polystyrene sulfonate; PVK: polyvinyl carbazole; TAZ: 3-(4-Biphenylyl)-4-phenyl-5-tert-butylphenyl-1, 2, 4-triazole; TFB: Poly[(9,9-dioctylfluorenyl-2,70diyl)-co-(4-4’-(N-(4-sec-butylphenyl)) diphenylamine)]; TPBI: 1,3,5-tris(N-phenylbenzimidazole-2-yl)-benzene; TPD: N, N’-diphenyl-N, N’-bis(3-methylphenyl)-(1, 1’-biphenyl)-4, 4’-diamine; B: blue; G: green; Y: yellow; O: orange; R: red; W; white

### 5.2. Downconversion of Blue or Ultraviolet Light

**Figure 19:**
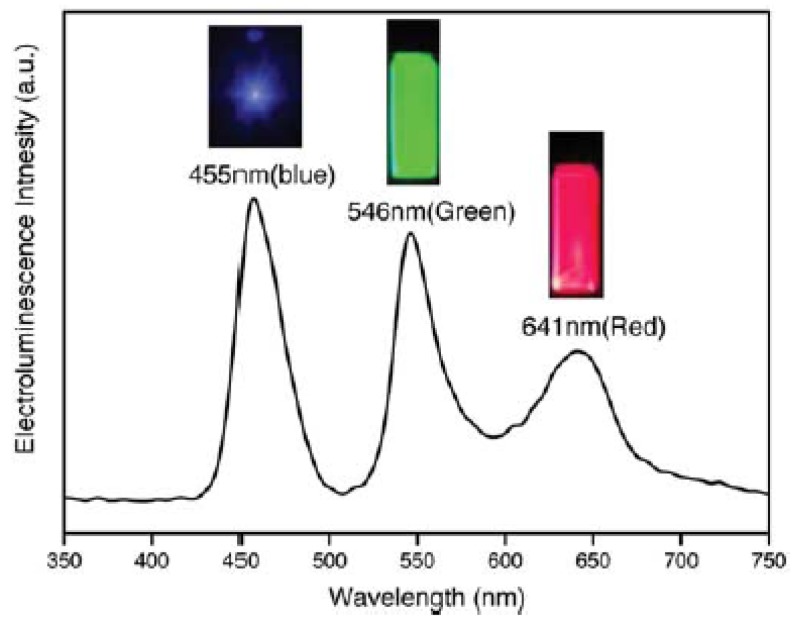
Downconversion of 455 nm blue emission from an InGaN light emitting diode to green and red by size-tuned CdSe/ZnSe Qdots in a silicone matrix (color rendering index: 91; chormacity coordinate: 0.33, 0.33) [reprinted with permission from [270] (© 2006 IEEE)].

Qdots are being used in downconversion of high energy to lower energy light. The advantages of Qdots phosphors over conventional inorganic phosphor and/or organic dyes include the (i) high QY of Qdots, (ii) emission range from UV to visible to NIR, (iii) better stability compared to organics, (iv) narrow FWHM ~30 nm (higher color saturation compared to conventional phosphor with typical FWHM: 50–100 nm), and (v) a large absorption window (band-gap to UV, enabling simultaneous excitation of different size Qdots). The main disadvantage of Qdots is self-absorption resulting from overlap of the absorption spectra from larger Qdots with the emission spectra and emission spectra from smaller Qdots. In 2000, *Lee et al.* [161] reported downconversion of 425 nm blue (from GaN based commercial LED) and UV light (from Hg lamp) using 2.0, 2.6, 4.6 and 5.6 nm CdSe/ZnS Qdots dispersed in a poly-aurylmethacrylate polymer matrix. The composite was prepared at 70–75 °C for 2 hrs by mixing as-synthesized TOP capped CdSe/ZnS Qdots and monomer of 1-aurylmethacrylate, then adding ethyleneglycol dimethacrylate and a radical initiator azo-bis-isobutyronitrile for crosslinking. Recently, organics-capped ZnSe Qdots [196] were excited with a near-UV InGaN LED to produce white light with *Commission Internationale de l’Eclairage* (CIE) x, y chromaticity coordinates of 0.38, 0.41. Downconversion of blue light (455 nm) from an InGaN LED resulted in white light emission with a color rendering index (CRI) of > 90 and CIE of 0.33, 0.33, when a mixture of green and red emitting CdSe/ZnSe Qdots was used [270], as shown in Figure 19. We also summarized some of the literature reports on downconversion of Qdots in Table 8. 

**Table 8:** Selected reports on the use of Qdots to downconvert blue or UV light from inorganic LEDs.

Year	Source light	Qdots	Matrix	Emitted light	Ref.
2000	UV (Hg lamp), Blue GaN Commercial LED	CdSe/ZnS (2.0, 2.6, 4.6, 5.6 nm)	Polyauryl-methacrylate	UV: Blue, orange, red; Blue: red (590 nm)	[161]
2005	InGaN (near UV)	ZnSe (TOPO & Stearic acid coated)	organics coated ZnSe (10 wt%) dispersed in epoxy resin	White; CIE (0.38, 0.41) Conversion efficiency: 30% relative to RGB commercial phosphors	[196]
2006	InGaN (455 nm)	CdSe/ZnSe (G); CdSe/ZnSe (R) CdSe/ZnSe (Y)	TOPO-coated CdSe/ZnSe dispersed in silicone	White, CIE: (0.33, 0.33), CRI: 91 with R& G; White, CIE: (0.32, 0.33), CRI: 50 with Y; Efficiency: 15-30 lm/W	[270]
2007	390 nm UV LED	CdSe/CdS/ZnS	2wt% Qdot in chloroform & epoxy resin at 1:1 (vol); Thermally cured	Red (620 nm)	[168]
2007	InGaN/GaN (440 nm, 452 nm)	CdSe/ZnS (440-452	Qdots blended with resin; 400 -1700 μm (Qdot density: 3.04-140 nanomoles/1ml resin)	White; with 453 nm & CdSe/ZnS (540, 500, 580 & 520 nm): CIE (0.24, 0.33), CRI: 71	[271]
2008	InGaN/GaN (blue/green)	CdSe/ZnS (620nm, R) & Au particles (for surface Plasmon enhanced emission)	5 wt% Qdots and 0.05 wt% Au in toluene spin-coated on LED (thickness ~200 nm)	White: (0.27, 0.24); Conversion efficiency ~53%	[272]

R: red, G: green, B; blue, O: orange; Y: yellow; W; white; CIE: International Commission on illumination; CRI: color rendering index; vol.: by volume

### 5.3. Quantum Dots in Solar Cell Device Fabrication

Increasing demands of energy for human activity warrant immediate attention, and solar energy is clean and plentiful. Approximately, a 9 ° 10^22^ J of energy reaches the earth everyday from the sun, compared to daily consumption by mankind of about 9 ° 10^18^ J [273]. Although the maximum thermodynamic efficiency (*Shockley-Queisser* limitation) from a single junction silicon solar cell is only about 31% [274], a multi-junction cell, that uses multiple subcell band-gaps to divide the broad solar spectrum into smaller segments, can exhibit higher for theoretical conversion efficiencies: e.g., ~40% for three and >58% for four or more subcells [275]. In fact, *King et al.* reported an efficiency of 40.7% from a three-junction GaInP/GaInAs/Ge cell under the standard spectrum for terrestrial concentrator solar cells at 240 suns [275]. Below, the benefits of using Qdots to fabricate inorganic-organic hybrid solar cells are discussed. 

**Figure 20:**
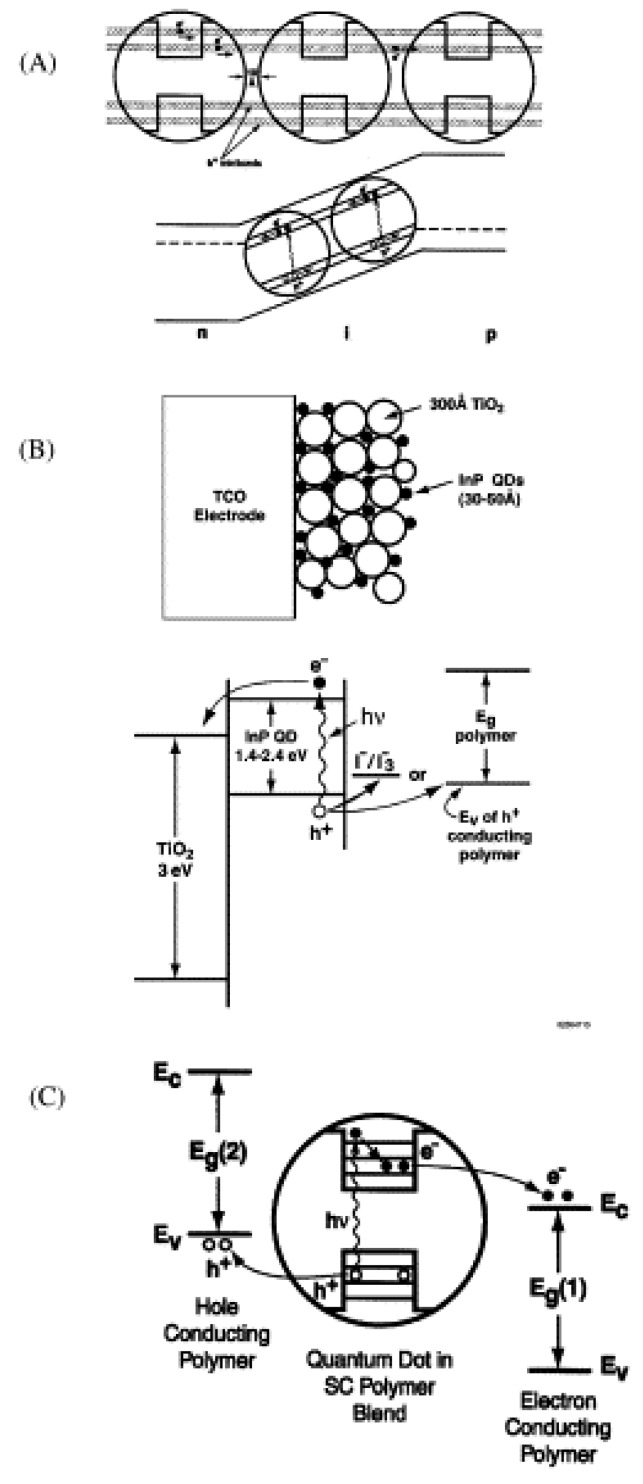
Solar cell with Qdots can be broadly classified in three categories: (A) P-I-N solar cell with a Qdots array, (B) Qdot sensitized and (C) Qdots dispersed [reprinted from publication [290], Copyright 2002, with permission from Elsevier].

A major factor leading to the superior efficiency of inorganic devices compared to organic or hybrid devices is the difference in carrier mobilities. Organic semiconductors possess relatively high absorption coefficients (usually ≥ 10^5 ^cm^-1^) [276]. In a solar cell, four consecutive processes occur: (1) absorption of light and exciton formation, (2) exciton diffusion, (3) charge separation, and (4) charge transportation. Due to poor mobility and a short exciton lifetime in conductive polymers, organics possess low exciton diffusion length (10–20 nm). In other words, excitons that form far from the electrode or carrier transport layer recombine and conversion efficiency decreases. For example, single and multiwall carbon nanotubes (SWNT and MWNT) were linked to CdTe Qdots capped with thioglycolic acid to produce solar cells [277]. The nanotubes provided a good hole transport route to the electrode, and CdTe acted as an excited-state electron donor. The highest monochromatic photon quantum efficiency was 2.3% from a hybrid cell consisting of single SWNT/pyrene+/red-emitting CdTe Qdots. Therefore, the combination of organic and inorganic materials improved the conversion efficiency of the solar cell. Hybrid inorganic/organic hybrid solar cells have been reported using CdSe [278], TiO_2_ [279], ZnO [280], PbS [281], PbSe [282], CuInS_2_ [283] and CuInSe_2_ [284]. Use of Qdots in solar cell is advantageous because: (i) quantum confinement enables size-tuned tunable band-gaps so that a multi-junction device is possible using the same Qdot composition to absorb entire gamut of sunlight from UV to visible to IR (0.5 eV to 3.5 eV); (ii) hot carrier relaxation dynamics can be significantly reduced due to confinement of exciton; (iii) multiple exciton generation or MEG [105,182,285,286,287,288,289] (e.g., IV-VI, Si, InAs Qdots) with a single photon is possible (*i.e.,* a QY >100%); (iv) good heterojunction with hole conductors are possible; (v) they are more stable and resistant to oxygen, moisture and UV radiation as compared to polymer (vi) lower-cost solution based fabrication processes can be used; (vii) substrate can be flexible. In general, Qdot based solar cells can be classified into three categories [285,290], as shown in Figure 20. 

#### 5.3.1. Quantum Dot Sensitized Solar Cell

**Figure 21:**
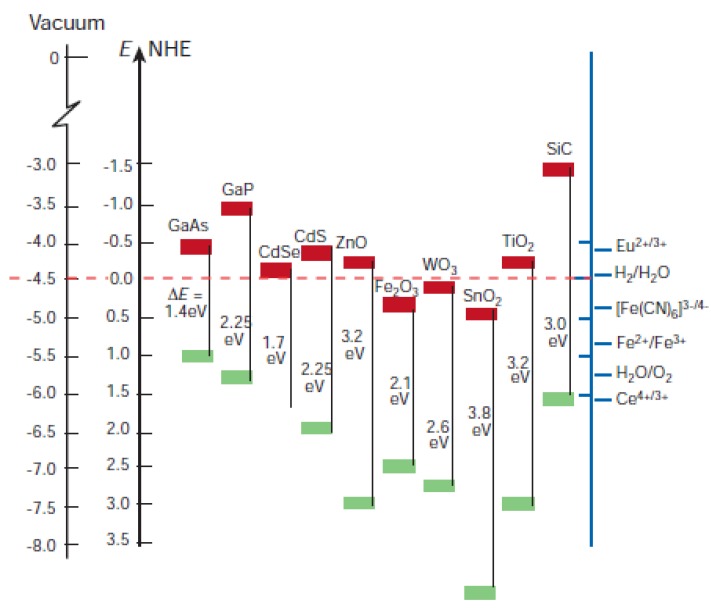
Valence and conduction band energies of various semiconductors, which have been used in solar cells. The outer left ordinate shows the energies referenced to the vacuum level, while the inner scale shows the normal hydrogen electrode scale (both in eV). (Reprinted by permission from Macmillan Publishers Ltd. [Nature (London)] [292], Copyright 2001).

For several decades, dyes have been used as sensitizers for harvesting energy. In the 1990s, the dye-sensitized solar cell (DSC) was extensively developed by *Graetzel* [291,292]. In a DSC, light is absorbed by the dye followed by an electron transfer from an excited state of the dye molecule into the conduction band of a wide band-gap semiconductors [292,293], such as TiO_2_, ZnO, TiO_2_, NbO_2_ or Ta_2_O_5_. Figure 21 shows the band position of some of the semiconductors that are used in photochemical cells. The hole on the dye is scavenged by a redox couple in solution. For better efficiency and charge separation, less than a monolayer of dye is required on the wide band-gap semiconductors. Usually dyes have a strong absorption band in the visible region but very low absorption in the UV and NIR regions. In addition, dyes in DSC suffer from photodegradation. Therefore, tunable band-gap semiconductors are of interest for the fabrication of sensitized solar cell. For example, the absorption of Qdots can be tuned from UV through the visible into the NIR, and band-edge absorption is favorable for effective light harvesting [293]. Finally, surface passivation can enhance photostability of Qdots. Selected results for Qdots sensitized solar cell are summarized in Table 9. 

Sensitization of highly porous TiO_2_ electrode by *in situ* synthesized CdS Qdots (4–20 nm) was demonstrated in 1990 [294,295]. After further development, a photocurrent QY of 80% with V_OC_ up to 1 V was measured using PbS, CdS, Ag_2_S, Sb_2_S_3_ and BiS_3_ Qdots as sensitizers [293]. Recently, organic hole conductor layers are introduced have replaced solution in electrochemical cells. In other words, research on electrochemical solar cells is moving towards heterojuction-based solar cell. *Plass et al.* [296] demonstrated a high surface area *p-n* heterojunction solar cell, where the heterojunction was sensitized to visible light by PbS Qdots. By functionalizing the surface, self assembled Qdots [297,298,299,300] on titania were used to improve the efficiency of solar cell. *Kamat’s group* [299] used different bifunctional linker molecules to assemble Qdots on TiO_2_ particles. *Niitsoo et al.* [300] reported an efficiency of 2.8% from a self-assembled monolayer of CdSe or CdS Qdot (6–8 nm) on titania using chemical bath deposition technique. A similar method was used by *Lin et al.* [298] and they achieved an efficiency of ~1.4%. Using TiO_2_ inverse opal and Qdots, *Diguna et al.* [301] achieved a power conversion efficiency of 2.7%. *Sun et al.* [302] used TiO_2_ nanotubes arrays and CdS Qdots for photoelectrochemical solar cell. Under AM 1.5 G illumination, the conversion efficiency was found to be 4.15%. Titania nanotubes were produced by anodic oxidation [303]. Recently, a conversion efficiency of > 4% was reported [304] when a ‘cascade’ energy level structure of TiO_2_/CdS/CdSe/ZnS was used. 

**Table 9:** Selected data for Qdot sensitized solar-cell.

Year	Qdots	Results	Conversion Efficiency %	Device Structure	Ref
1994	CdS: 4nm PbS: 5nm	J_SC_: ~1.9 mA/cm^2^ V_OC_: 1 V FF: 0.45 EQE: 80% (@ 460 nm)		ITO//TiO2//CdS or PbS	[293]
1998	InP: 6.5 nm	FF: 0.685	1.5% (400–800 nm)	TiO2//InP	[305]
2002	PbS: 7 nm	V_OC_: 0.24V EQE: 45%	(at AM 1.5) 0.49%	SnO2:F//TiO2//PbS//Spiro-OMeTAD, Au	[296]
2006	CdSe CdS ~5nm	J_SC_: 10.5 mA/cm^2^ V_OC_: 0.66 V FF: 39.5	2.8%	TiO2//CdSe or CdS (self-assembled)	[300]
2007	CdS (4–6 nm)	J_SC_: 3.44 mA/cm^2^ V_OC_: 0.657 V FF: 0.6	1.35%	TiO2//CdS (Self-assembled)	[298]
2007	CdSe	J_SC_: 7.51 mA/cm^2^ V_OC_: 0.71 V FF: 0.5	2.7%	TiO2//F//CdSe//F//ZnS	[301]
2008	CdS	J_SC_: 7.82 mA/cm^2^ V_OC_: 1.27 V FF: 0.578	2.8%	TiO2 (nanotubes)//CdS	[302]
2009	CdS CdSe	FF: 0.49 V_OC_: 0.5137 V J_Sc_: 16.8 mA/cm^2^	4.22% (AM 1.5)	TiO_2_//CdS(3)//CdSe(4)//ZnS, Au	[304]

*AM: Air mass*; V_OC_: open circuit voltage; I_SC_: short circuit current; FF: Fill factor; OMeTAD: 2,2’,7,7’-tetrakis(N,N-di-p-methoxyphenyl-amine)9,9’-spirobifluorene.

#### 5.3.2. Quantum Dot Dispersed Solar Cell

In 1992, *Wang and Herron* measured the photoconductivity effect of Qdots using *in situ* synthesized CdS Qdots in poly(vinyl-carbazole) matrix with Al electrode [306]. Greenham* et al.* fabricated an inorganic-organic hybrid solar cell using a mixture of 5 nm CdSe Qdot in MEH-PPV polymer that was spin-coated onto an ITO/glass substrate and an Al cathode was deposited to complete the device. The energy conversion efficiency under monochromatic illumination at 514 nm (5 W/m^2^) was 0.2% [307]. Because organic semiconductor materials generally exhibit low electron mobilities (below 10^-4^ cm^2^V^-1^s^-1^), mixing them with inorganic nanostructured semiconductors can result in improved device performance [308]. *Huynh et al.* [309] improved the device performance by incorporating Qdots and nanorod in poly-3(hexylthiophene) (also known as P3HT). Incorporation of Qdots resulted in a maximum EQE of 55% with a power conversion efficiency of 1.7%. ZnO Qdots have been used since they are *n*-type semiconductors with high carrier concentrations and electron mobilities [310]. Hybrid solar cells based on ZnO Qdots under an AM1.5 illumination exhibited an energy conversion efficiency of 0.9% for P3HT, 1.6% for poly[2-methoxy-5-(3’,7’-dimethyloctyloxy)-1,4-phenylene vinylene] (MDMO-PPV), and EQE of 27% at 480 nm for P3HT and 40% for MDMO-PPV. The device structure was ITO/ PEDOT:PSS/ZnO: polymer (26 vol%, thickness ~200nm)/Al. Thermal annealing of the spin cast ZnO nanoparticles/polymer blend improved charge transport and was crucial to achieving the efficiencies reported above. Note that the above discussion emphasizes results mainly from spherical semiconducting nanoparticles. There are reports on the use of nanorod [311,312], tetrapod [313,314,315] and highly brunched [316] nanoparticles in solar cells. Selected results from Qdots-based solar cells are summarized in Table 10. 

**Table 10:** Selected results for solar cells using Qdot dispersed in a conducting polymer.

Year	Qdot Size	Results	Conversion Efficiency % (at AM 1.5 G)	Device Structure	Ref
1997	5nm	V_OC_: 0.5 V FF: 0.26 EQE: 12%	0.2 (monochromatic illumination at 514 nm)	ITO// MEHPPV: CdSe or CdS//Al	[307]
2002	CdSe or CdS Dot: 7nm Rod: 7 X 60 nm	V_OC_: 0.7V FF: 0.4 EQE: 55% (20% Qdots)	1.7	ITO//CdSe: P3HT//Al	[309]
2004	CdSe: Rod: 7 x 30 nm		1.5	ITO//PEDOT:PSS//P3HT-functionalized CdSe //Al	[317]
2006	PbS: 4nm	V_OC_: 1 I_SC_: -0.13 FF: 0.28	0.7	ITO//PEDOT:PSS//PbS:MEHPPV//Al	[318]

FF: Fill factor; I_SC_: short circuit current; ITO: indium tin oxide; MEHPPV: poly[2-methoxy-5-(2-ethylhexyloxy)-1,4-phenylenevinylene]; PEDOT:PSS: poly(3,4-ethylene-dioxy-thiophene) poly(styrene-sulfonate); P3HT: poly-3(hexylthiophene); V_OC_: open circuit voltage.

### 5.4. Quantum Dots in Other Optoelectronic Devices

Qdot photonic devices, including optical amplifiers and lasers operating at room temperature, have been studied [19,319,320]. A major difficulty in achieving lasing is the very efficient nonradiative *Auger* recombination processes. Several strategies have been proposed to improve the lasing, including increased Qdot concentrations in the active layer, use of improved optical feedback structures, and optimized optical waveguides. Two dimensional waveguides loaded with luminescent colloidal CdTe Qdots grown with a layer-by-layer deposition technique have been investigated [321]. The waveguides exhibited propagation loss coefficients of <1 cm^−1^. The losses depended weakly on the width of the waveguides but depend strongly on the surface roughness. High optical gain of ~230 cm^−1 ^was demonstrated using femtosecond pulses [321], showing that this kind of Qdot waveguides could be suitable for lasers and optical amplifiers. Qdots embedded composites were also used to demonstrate photorefractivity and other nonlinear optoelectronic properties [222,322]. PbSe Qdots in a polymeric host showed large photorefraction, photoconductivity and optical gain when excited with a low-power, continuous-wave laser at 1550 nm [323]. Dynamic-photorefractive holographic gratings were written in the composite. The net optical gain and significant diffraction efficiency achieved with the low power laser makes these nanocomposites potential choices for infrared imaging and optical communication applications.

III-V semiconductors, such as InGaAlN, GaP, GaAs, InP and InAs, are very important for the development of optoelectronic devices as it is possible to engineer III-V Qdots simply by tuning the Qdot size and composition to emit anywhere from the IR to the UV [324,325]. Nitride-based Qdots have attracted enormous research interest because of their large built-in electric fields [324]. These large fields originate from both spontaneous polarization in the wurtzite crystal structure, as well as the lattice-mismatch strain acting through their large piezoelectric constants. Emission from InAs/InP Qdots can be controlled by size as well as the As and P concentrations, and are important for fiber optic telecommunication systems [326]. InP-based material can emit over the range of wavelengths necessary for photonic devices, especially near 1.55 μm. InAsP Qdots are suitable for monolithically integrated photonic devices on a single chip, including lasers, phase modulators, detectors, and passive waveguides. 

### 5.5. Application of Quantum Dots in Bioimaging Applications

**Figure 22:**
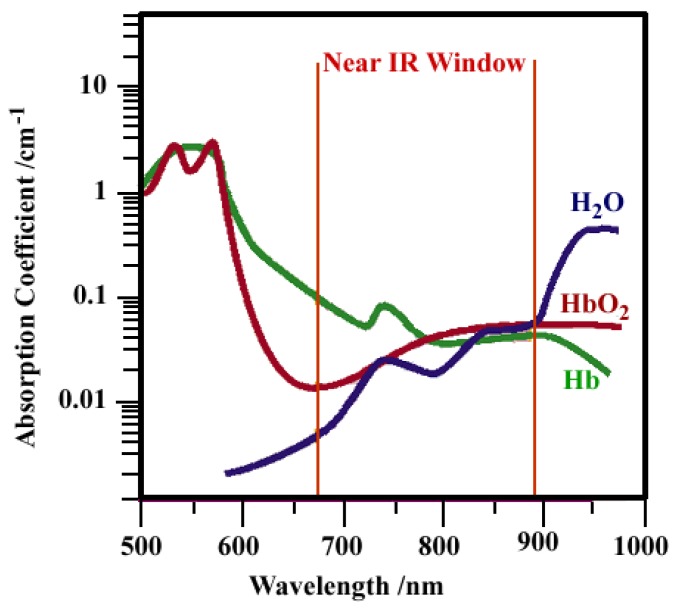
Absorption *versus* wavelength for hemoglobin and water showing the near infrared (NIR) window for *in vivo* imaging to minimize absorption and scattering [346].

Currently, magnetic resonance imaging (MRI), optical imaging, and nuclear imaging are emerging as key imaging techniques in biological systems [327]. They differ in terms of sensitivity, resolution, complexity, acquisition time and operational cost. However, these techniques are complementary to each other most of the times. There are several reviews on the physical basis of these techniques [327,328], the instrumentation [329,330] and the issues that affect their performance [331,332]. Currently, a significant amount of research is aimed at using the unique optical properties of Qdots in biological imaging [23,58,59,92,332,333,334,335,336,337,338,339,340,341,342]. Much of optical bioimaging is based on traditional dyes [343,344], but there are several drawbacks associated with their use. It is well-known that cell autofluorescence in the visible spectrum [345] leads to the following five effects. (1) The autofluorescence can mask signals from labeled organic dye molecules. (2) Instability of organic dye under photo-irradiation is well known in bioimaging which results in only short observation times. (3) In general, conventional dye molecules have a narrow excitation window which makes simultaneous excitation of multiple dyes difficult. (4) Dyes are sensitive to the environmental conditions, such as variation in pH. (5) Most of the organic dyes have a broad emission spectrum with a long tail at red wavelengths which creates spectral cross talk between different detection channels and makes it difficult to quantitate the amounts of different probes. Qdots, on the other hand, are of interest in biology for several reasons, including (1) higher extinction coefficients; (2) higher QYs; (3) less photobleaching; (4) absorbance and emissions can be tuned with size; (5) generally broad excitation windows but narrow emission peaks; (6) multiple Qdots can be used in the same assay with minimal interference with each other; (7) toxicity may be less than conventional organic dyes, and (8) the Qdots may be functionalized with different bio-active agents. In addition, NIR-emitting Qdots can be used to avoid interference from the autofluorescence, since cell, hemoglobin and water have lower absorption coefficient and scattering effects in the NIR region (650 – 900 nm) (see Figure 22). Light is routinely used for intravital microscopy, but imaging of deeper tissue (500 μm – 1 cm) requires the use of NIR light [346]. Inorganic Qdots are more photostable under ultraviolet excitation than organic molecules, and their fluorescence is more saturated. 

Among nanostructured materials, Qdot-based size-tuned emission color offers the potential to develop a multicolor optical coding technique, e.g., by functionalizing different sized CdSe Qdots with different molecules. Researchers have used Qdots for *in vivo* and *in vitro* imaging and diagnostic of live cell as a complement to or replacement of conventional organic dyes [353,354,355]. Some of the studies are tabulated in Table 11. Exploiting the properties of Qdots, such as (1) sharp and UV-NIR tunable fluorescence, (2) charge transfer through fluorescence resonance energy transfer (FRET), (3) surface enhanced Raman spectroscopy (SERS) (4) Radio opacity and paramagnetic properties, and/or (5) MRI contrast agent, it has been shown that Qdots can be used for bioimaging purpose. In the following section, we briefly discuss these imaging techniques that are reported in literature.

**Table 11:** Selected *in vitro* and *in vivo* bioimaging studies using Qdots.

Qdots	Purpose	Imaging Techniques	Emission/ Size of Qdots	Ref
CdSe/CdS/SiO_2_	Mouse fibroblast cell imaging	*In vitro* Fluorescence	550 nm & 630 nm	[347]
CdSe/ZnS	Biological detection/ sensing	*In vitro* fluorescence	1–4 nm	[348]
CdSe/ZnS/SiO_2_	Phagokinetic track imaging	*In vitro* fluorescence	554 nm & 626 nm	[349]
CdSe/ZnS	Tumor vasculature and lung endothelium imaging	*In vitro* and *in vivo* fluorescence	<10 nm	[350]
CdTe/CdSe	Cancer cell lymph nodes imaging	*In vivo* Fluorescence	NIR	[351]
CdSe/ZnS	Maltose binding Protein	*In vitro* FRET	560 nm	[352]

FRET: Fluorescence resonance energy transfer; NIR; near infrared.

#### 5.5.1. Fluorescence for Bioimaging

**Figure 23:**
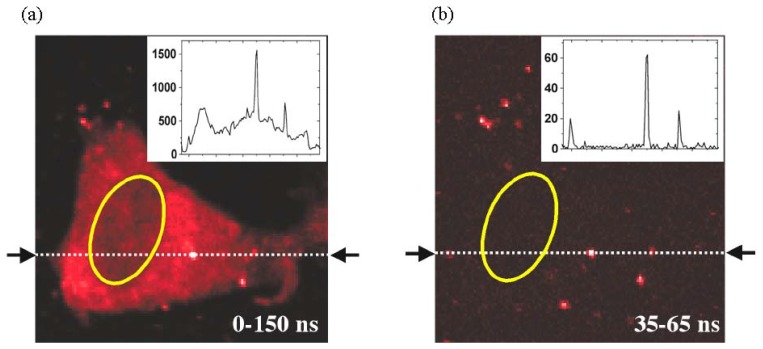
Time-resolved confocal optical micrography of 3T3 cell. (a) A micrograph acquired from all the detected photons; (b) Time-gated micrograph constructed from only photons that arrived 35–65 ns after the laser pulse (laser intensity: 0.1 kW.cm-2; integration time per pixel: 25 ms. Inset figures show cross-sections along the same horizontal line (indicated by the black arrows) for (a) and (b) [360] (Copyright Optical Society of America).

Qdots fluorescence-based bioimaging [356,357,358] can be broadly classified into four types of modes: intensity, spectrum, lifetime and time-gated. All of these modes can be used at the same time for multimodality imaging. Generally, a high QY from Qdots is required for intensity-based imaging. On the other hand, their narrow emitting spectra make Qdots suitable for multiple colors imaging. The longer fluorescence lifetime of Qdots compared with that of tissue avoid the noise from autofluorescence. Therefore, there is an advantage to use both lifetime and time-gated modes simultaneously (Figure 23). PL from Qdots has been a widely used tool in biology. In 1998, *Alivisatos* and his group showed that the Qdots were potential candidates for biological applications [347]. To establish the use of Qdots, biotin was covalently bound to the Qdot surface and used to label fibroblasts, which was incubated in phalloidin-biotin and streptavidin.

For biological and medical applications, it is of importance to study the photophysical properties of Qdots in living cells [359], particularly photo-induced optical properties of the intracellular Qdots. After injecting thiol-capped CdTe Qdots into living cells, the PL intensity increased with time and the emission peak blue-shifted [359]. De-oxygenation prevented the PL blue shift, suggesting that photoactivated oxygen was responsible. The activated oxygen is presumably formed from the oxygen that intercalates the thiol layer at the Qdot core surface. When Qdots are used as fluorescence probes for cellular imaging, the effects of the PL blue shift and photobleaching must be considered.

Spectral encoding Qdot technology [338,361] is expected to open new opportunities in gene expression studies, high-throughput screening, and medical diagnostics. The broad absorption spectra of the Qdots allow single wavelength excitation of emission from different-sized Qdots. Multicolor optical coding for biological assays has been achieved by using different sizes of CdSe quantum dots with precisely controlled ratios. The use of 10 intensity levels and 6 colors could theoretically encode one million nucleic acid or protein sequences. *Nie et al.* embedded different-sized Qdots into highly uniform and reproducible polymeric microbeads, which yielded bead identification accuracies as high as 99.99% [362]. 

The luminescent lifetime of CdSe Qdots (several tens of nanoseconds) is longer than that of cell autofluorescence (~1 ns), which permits measurement of marker spectra and location without high backgrounds through the use of time-gated fluorescent spectroscopy and/or microscopy. In addition, the photostability of CdSe is much better than that of conventional organic dyes [363], allowing data acquisition over long times with continuous excitation. Compared with conventional organic dyes, Qdots have longer lifetime allowing acquisition of low background PL images by using time-gated fluorescent microscopy, as shown in Figure 22 [360]. In an another study [364] CdSe Qdots-based deep tissue imaging of the vasculature system was carried out highlighting various internal structures. The mice used in this study showed no ill effects from the Cd-containing labels. 

**Figure 24:**
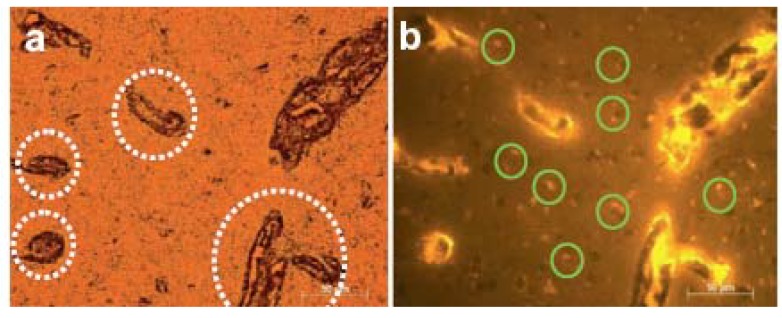
(a) Transmission optical (b) fluorescence microscopy images (40X) of cross-section of fixed brain tissue showing luminescence in (b) from CdS:Mn/ZnS core/shell Qdots [93] – Reproduced by permission of The Royal Society of Chemistry (hyperlink to the article: http://dx.doi.org/10.1039/b503234b).

In order to enhance the lifetime of the emission, some transition or rare earth elements are intentionally incorporated into the Qdots. These activators create local quantum states that lie within the band-gap and provide states for excited electrons or traps for charge carriers and result in radiative relaxations towards the ground state. For transition metal ions such as Mn^2+^, the lifetime of the luminescence [46,76,180] is on the order of milliseconds due to the forbidden *d-d* transition. *Santra et al.* [93,95,365] demonstrated *in vivo* bioimaging capability using amine modified Mn-doped CdS/ZnS core/shell Qdots conjugated to a TAT peptide (a cell penetrating peptide). Transmission optical and fluorescence micrographs (Figures 24(a) and (b)) of a cross-section of fixed brain tissue clearly showed the blood capillaries (broken white circle in Figure 24(a)) and surrounding brain cells. It was also shown that the TAT conjugated Qdots reached the nucleus of the brain cells (green-circled brown spots in Figure 24(a)). It is well known that the TAT peptide can rapidly translocate through the plasma membrane and accumulate in the cell nucleus [93]. The histological analysis of the brain tissue supports the fact that TAT-conjugated Qdots crossed the blood-brain-barrier, migrated to brain parenchyma and reached the cell nuclei. Endothelial cells in the blood capillaries were found heavily loaded with CdS:Mn/ZnS Qdots and appeared as bright yellow lines in Figure 24(b).

The use of NIR photons is promising for biomedical imaging in living tissue due to longer attenuation distances and lack of autofluorescence in the IR region. This technology often requires exogenous contrast agents with combinations of hydrodynamic diameter, absorption, QY and stability that are not possible with conventional organic dyes [351]. Qdots-based contrast agent offers these properties. In addition, the emission can be tuned to the NIR window (see Figure 22) either by controlling the size of the Qdots or by incorporating rare-earth activators. The emission of CdTe/CdSe Qdots can be tuned into the NIR while preserving the absorption cross-section. It was shown that a polydentate phosphine coating onto the Qdots made the Qdots water soluble, allowing them to be dispersed in serum. Injection of only 400 pmol of NIR emitting Qdots permitted real time imaging of sentinel lymph nodes that were 1 cm below the surface using an excitation power density of only 5 mW/cm^2^ [351].

#### 5.5.2. Use of Fluorescence Resonance Energy Transfer in Bioimaging

**Figure 25:**
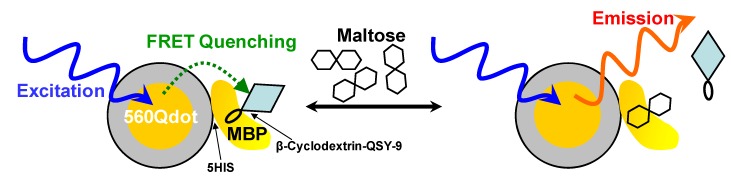
A schematic showing on the function of 560nm Qdot-MBP nanosensor. Each 560 nm emitting Qdot is surrounded by an average of ~10 MBP moieties (a single MBP is shown for simplicity). Formation of Qdot-MBP-β-CD-QSY9 (maximum absorption ~565 nm) results in quenching of Qdot emission. Added maltose displaces β-CD-QSY9 from the sensor assembly, resulting in an increase in direct Qdot emission (Reprinted by permission from Macmillan Publishers Ltd. [Nature Materials] [352] Copyright 2003).

Fluorescence resonance energy transfer (FRET) is a phenomenon in which photo-excitation energy is transferred from a donor fluorophor to an acceptor molecule. Based on *Förster* theory, the rate of this energy transfer depends on the spectral overlap of donor emission and acceptor absorption and the donor-acceptor spatial arrangement [366].The ability of Qdots to participate in FRET provides a mechanism for signal transduction in optical sensing schemes. CdSe Qdots can be used to build on-off switches by utilizing *Förster* resonance energy transfer between the Qdot donor and an organic acceptor. In such an optical sensing scheme, Qdots could act both as a donor and an acceptor. *Mattoussi and co-works* [367,368] have studied such an energy transfer between donor Qdots and acceptor dye molecules. In the blend of water-soluble CdSe/ZnS Qdots and maltose binding protein (MBP), MBP was assembled onto the surface of Qdots by both electrostatic self-assembly and metal affinity coordination (Figure 25). With increased fraction of MBPs, emission from the dye increased while that from Qdots decreased. In addition, the emission intensity from dye-labeled MBPs was dependent on the emitted color from different sized Qdots and the spectral overlap (as expected from *Förster* theory). *Mattoussi et al.* also investigated Qdots as an acceptor in a FRET process [369]. This on-off switch has the potential to be used as a sensor in many important applications, including healthcare, environmental monitoring and biodefense systems.

All of these experiments confirmed that water-soluble Qdots have potential applications in biosensor or bioimaging. FRET has been utilized to probe biological activity. *Patolsky et al.* reported that telomerization and DNA replication can be monitored with CdSe/ZnS Qdots [370]. As telomerzation proceeded, the emission from Texas-Red-labeled dUTP increased while that from quantum dots decreased *via* FRET. In replication studies, the Texas-Red dUTP was brought into close proximity of the nanocrystal, resulting in FRET from Qdots to organic dye. These results suggested the possibility of using Qdots in the detection of cancer cells or in amplification of DNA on chip arrays.

FRET coupled to quenching provided alternative path for sensing. The luminescence *via* FRET is turned on by the appearance of analyte, which displaces a quencher or a terminal energy acceptor. *Mattoussi et al.* have developed a sensor for maltose by adapting their CdSe-MBP conjugates for analyte displacement strategies [352]. First, a β-cyclodextrin conjugated to a non-fluorescent QSY9 quencher dye was docked to the MBP saccharide binding site of the CdSe/ZnS-MBP. Second, maltose displaced the β-cyclodextrin-QSY9 conjugate to restore quantum dots emission. This approach is general and the concept of an antibody fragment bound to a Qdot surface through noncovalent self-assembly should find wide use for other analytes of interest.

#### 5.5.3. Surface Enhanced Raman Spectroscopy

Surface enhanced Raman Spectroscopy (SERS) is a near-field probe which is sensitive to local environments. Qdot-based SERS can be used two ways in biomedical application, first as single molecule-based SERS that measures the unique fingerprint spectra of pure analytes on a Qdot. In a second approach, Qdots are covered by a monolayer of analyte, and SERS spectra are obtained from an ensemble of nanoparticles. In this case, population-average data are determined and one can get robust data from complex milieu or whole blood circulation.

By using the single molecule SERS technique along with Au/silica or core/shell Qdots, biomarker can be very sensitive. Currently, bioconjugated SERS have also been developed to identify protein biomakers on the surfaces of living cancer cells. For example, targeted gold nanoparticles are prepared by using a mixture of thiol*–*polyethylene glycol (PEG) and a heterofunctional PEG for live cancer cell detection, which binds to the epidermal growth factor receptor (EGFR) with high specificity and affinity [371]. Human head and neck carcinoma cells are EGFR-positive and can give strong SERS signals [372].

In contrast, the human non-small cell lung carcinoma does not express EGFR receptors, showing little or no SERS signals. Single cell profiling studies are of great clinical significance because EGFR is a validated protein target for monoclonal antibody and protein-kinase based therapies. In addition, Qdots-based SERS can be used for *in vivo* tumor targeting and detection. *Qian et al.* reported that they injected small dosage of nanoparticles into subcutaneous and deep muscular sites in live animals and highly resolved SERS signals were obtained [373]. It is estimated that the achievable penetration depth is about 1–2 cm for *in vivo* SERS tumor detection.

#### 5.5.4. Radio-Opacity and Paramagnetic Properties

CdS:Mn/ZnS core/shell Qdots were characterized for radio-opacity and magnetic hysteresis [94,95,365] for possible use as contrast agent in computer tomography (CT) and MRI due to electron dense Cd and paramagnetic Mn, respectively [94]. For radio-opacity, the Qdots sample was compared with a conventional radio-opaque dye, *Omnipaque*, used for CT scans and angiography. It was found that the X-ray absorption of Qdots was less than that of *Omnipaque*. In this respect, Qdot may not provide sufficient contrast for current radiographic practice. A superconductor quantum interface device magnetometer was used to measure the magnetization of CdS:Mn/ZnS Qdots. A typical room temperature hysteresis curve for paramagnetic CdS:Mn was observed, but it is too small for MRI imaging.

#### 5.5.5. Magnetic Resonance-based Bioimaging

MRI is essentially proton nuclear magnetic resonance (NMR) [332,374]. Protons are excited with short pulses of radio frequency radiation and the free induction decay as they relax is measured and deconvoluted by a *Fourier* transform, which provides an image of the tissue. Areas of high proton densities, e.g., water or lipid molecules, have a strong signal and appear bright. Areas of bone or tendon, which have a low proton density, have a weak signal and appear dark. A major limitation of MRI is its inability to distinguish between various types of soft tissues where the relative proton densities can be very similar. Regions having air pockets and fecal matter, such as the bowel, are hard to image because of inconsistent proton density. Therefore, various contrast agents such as perfluorochemicals, oils, fats and nanomaterials, have been studied to circumvent these imaging problems. Unlike organic molecules, nanomaterials-based contrast agents are miscible in aqueous systems which allow them to be used intravenously. Therefore, they are well suited for *in vivo* applications such as tracking blood flow in the brain. Another advantage of Qdots over other nanoparticles is that they offer multimodal imaging capabilities [83]. However, appropriate functionalization of the Qdots is needed in order to make the Qdot a suitable contrast agent for MRI.

**Figure 26:**
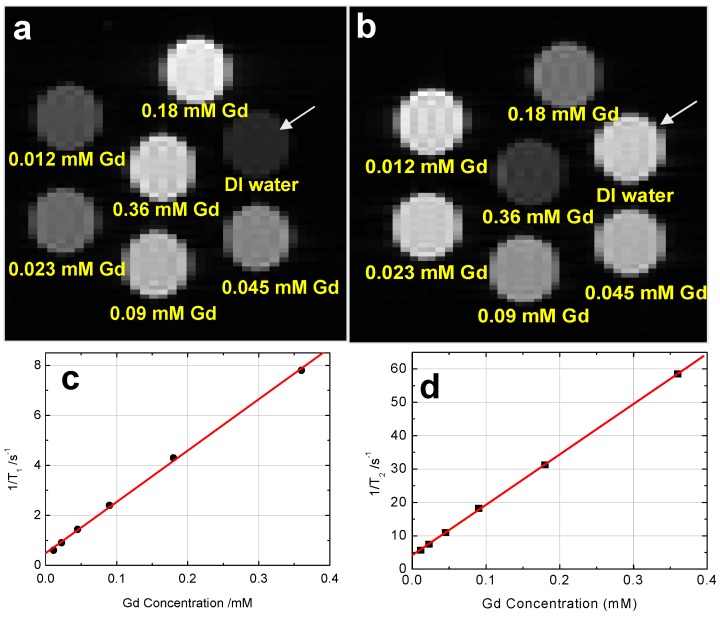
Magnetic resonance data from Gd-functionalized silica-coated CdS:Mn/ZnS Qdots: (a) T_1_-weighted (repetition time (T_R_) = 11000 ms, echo time (T_E_) = 4.2 ms), (b) T_2_-weighted (T_R_ = 11000 ms, T_E_ = 24 ms) images of deionized (DI) water and serial dilutions of Gd-functionalized Qdots (0.36, 0.18, 0.09, 0.045, 0.023, 0.012 mM of Gd). Linear plots of Gd concentration *versus* 1/T_1_ (c) and 1/T_2_ (d) to obtain ionic relaxivities of R_1_ and R_2_ of Gd-Qdots [reproduced with permission from [69]. Copyright Wiley-VCH Verlag GmbH & Co. KGaA].

Qdot-based contrast agents change the strength of the MRI signal at a desired location. For example, paramagnetic contrast agents change the rate at which protons decay from their excited state to the ground state, allowing more rapid decay through energy transfer to a neighboring nucleus [374]. As a result, regions containing the paramagnetic contrast agent appear darker in an MRI than regions without the agent. When paramagnetic Qdots are delivered to the liver, the uptake rate of Qdots by healthy liver cells is much higher that by diseased cells. Consequently, the healthy regions are darker than the diseased regions. Several experimental reports in the literature demonstrate the usefulness and multi-modal use of Qdots in MRI applications [69,375,376,377,378]. In these reports, the Qdots are often coated with a water soluble paramagnetic coating to enhance contrast. *Yang et al.* [69] synthesized a water soluble Gd-functionalized silica coated CdS:Mn/ZnS Qdots and studied them a for MRI contrast agent. Longitudinal (T_1_) and traverse (T_2_) proton relaxation times were measured with a single slice, spin-echo image sequence at 4.7 Tesla. Increased magnetic resonance (MR) signal intensity, as shown in Figure 26(a), was observed [69,95,365] with increasing Gd concentrations due to the shorter water relaxation time T_1_. In T_2_ weighted images, the MR signal intensity was substantially decreased by the effects of increased Gd on the T_2_ of water (Figure 26(b)). For control experiments, T_1_ and T_2_ weighted images of serial dilutions of Qdots without Gd^3+^ ions were recorded and could not be distinguished from those of deionized (DI) water.

Normalized T_1_ and T_2_ weighted intensities *versus* repetition time (T_R_) and echo time (T_E_), respectively, for DI water and a series of diluted Gd-functionalized Qdots (from 0.36 to 0.0012 mM of Gd) showed increasingly faster recovery of longitudinal magnetization and faster decay of transverse magnetization for increased Gd concentrations [69,95,365], as shown in Figure 26(c) and Figure 26(d) . The efficacy of a contrast agent is generally expressed by its relaxivity (R_i_), that is defined by 1/*T_i_* = 1/*T^o^* + *R_i_*[Gd], [379], where T_i_ is the relaxation time for a contrast agent solution concentration of [Gd], and T^o^ is the relaxation time in the absence of a contrast agent. The relaxivities R_1_ and R_2_ were found to be 20.5 and 151 mM^-1^s^-1^, respectively. Compared to commercially available contrast agents, Gd-functionalized Qdots exhibited higher R_1_ and R_2_ values under the same magnetic field strength of 4.7 T [380]. High relaxivities were attributed to a reduced tumbling rate of the Gd^3+^-based contrast agents by grafting the contrast agent to rigid macromolecules and avoiding free rotation of the chelate [381,382]. Although the Gd-Qdots can serve as either a T_1_ or T_2_ contrast agent, the R_2_/R_1_ ratio of ~7.4 indicates that they may be most effective as a T_2_ contrast agent.

Recent advances in use of Qdots in biology studies support the promise of a quantum leap in the extensive use of Qdots in future biological applications. It is predicted that Qdots will be able to provide unprecedented sensitivity and selectivity over the traditional practices for molecular imaging. The use of Qdots emitting in the NIR region will provide greater sensitivity and the longer lifetime of their excited states (as compared to organic fluorophores and proteins) will lead to for improved bioimaging. Despite the advantages for Qdots-based bioimaging, several issues related to Qdots need to be addressed before *in vivo* use, especially their toxicity. *Son et al.* [383] reported an ion exchange at the surface of CdSe Qdots that suppressed their PL intensity and led to the release of Cd^2+^, which is known to be toxic to human. The only way to partially recover the PL emission was to add excess Cd^2+^, which is unacceptable for biological application. In addition, bare Qdots were reported to be cytotoxic [384]. Some of the Qdots properties are limiting, such as size of Qdots, which sometimes is larger than the traditional organic marker dyes. As research on nanoparticles with novel properties continues, it should be possible to overcome these drawbacks and to develop multifunctional, multimodal Qdot-based systems for better biological imaging.

## 6. Perspective

**Figure 27:**
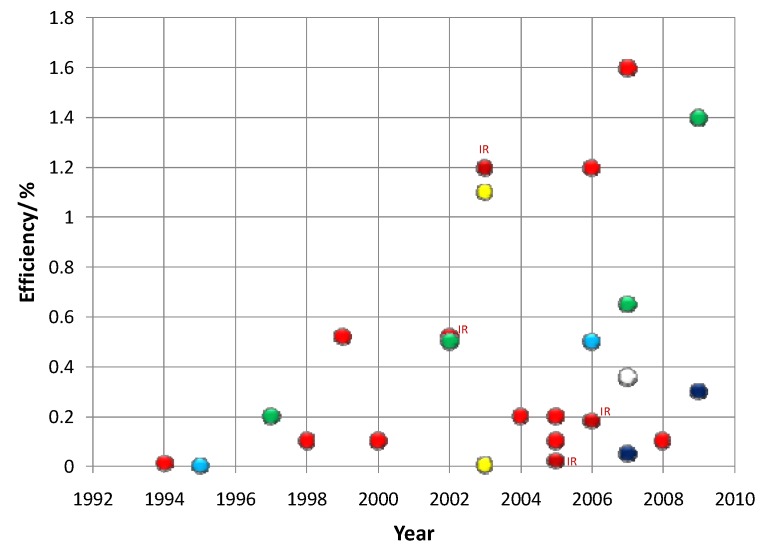
Efficiency of Qdot-based light emitting diode (QLED) *vs.* year reported in the literature; Colors of the legends represent the emitted color from the QLED (red: 601–700 nm; yellow: 561–600; green: 531–560 nm; sky blue: 491–530 nm; blue: 440–490 nm; IR: infrared emitting QLEDs are marked with IR; white: white emitting QLED).

In this review, selected aspects of the structure, properties, application and performance of Qdots have been discussed. Among the various branches in nanotechnology, these zero dimension nanostructures have paved the way for numerous advances in both fundamental and applied sciences. This is due to the fact that the Qdot exhibit significantly different optical, electronic and physical properties as compared to bulk materials. With respect to synthesis of Qdots, significant progress has been made in studies of the growth kinetics through both theoretical models and experimental data. Procedures ranging from simple wet chemical methods to very sophisticated and equipment-intensive atomic layer deposition techniques are being used to synthesize Qdots. The ‘bottom-up’ approaches are being widely explored resulting in a wider variety of methods to generate Qdots. Despite the large amount of research, there is still a lot to understand about the use of Qdots in large scale biological and solid-state optical applications, as we discussed throughout the article. For example, Qdot-based LED showed EQE ~1.5% (see Figure 27). It was also demonstrated that a QLED-based display is possible. Although preliminary experiments at the laboratory level have been successful, scaling up the production and retaining the properties of Qdots is not trivial. Continued research and development on Qdots will provide further improvements in quantum efficiency, better device fabrication and new materials which extend the emission into the near IR region.

Nanobiotechnology is predicted to be a major R&D area for this next century. However several stumbling blocks need to be overcome to ensure a close marriage of biology and nanotechnology. Bio-functionalizing the Qdots and interface engineering to control when electronic can pass are major challenges. Several applications, like displays, lighting, selective sensors, bio-imaging, MRI contrast agents, and bio-labels, need attentions to improve further and to answer questions about Qdot synthesis, properties, ageing and toxicity from the scientific and engineering community.

## Abbreviations

Ac: Acetate
ADF: Annular dark field
ALE: Atomic layer epitaxy
Alq3: Tris-(8-hydroxyquinoline) aluminum
AM: Air mass
AO: Atomic orbital
AOT: Aerosol OT
B: Blue
BCP: Bathocuproine
BSA: Bovine serum albumin
CBP: 4,4’,N,N’-diphenylcarbazole
CBP: 4,4’-N,N’-dicarbazolyl-biphenyl
CIE: International commission on illumination or Commission Internationale de l’Eclairage
CNPPP: Poly(2-(6-cyano-6’-methylheptyloxy)- 1,4- phenylene)
Co.sol: Coordinating solvent
CRI: Color rendering index
CT: Computer tomography
CTAB: Cetyl trimethyl-ammonium bromide
CV: Cyclic voltametry
CVD: Chemical vapor deposition
dBSA: Denatured bovine serum albumin
DDA: Dodecylamine
DEPE: 1,2-bis(diethyl-phosphino)-ethane
DI: deionized
DMPA: 2,2 –dimethoxy-2-phenylacetophenone
DOS: Density of states
DSC: Dye-sensitized solar cell
E_a_: Electron affinity
EELS: Electron energy loss spectroscopy
EGDMA: Ethylene glycol domethacrylate
EL: Electroluminescence
EMA: Effective mass approximation
EML: Emitting layer
EQE: External quantum efficiency
ERFR: Epidermal growth factor receptor
ESR: Electron spin resonance
Et: Ethyl
ETL: Electron transport layer
F6BT: Poly[(9,9-dihexylfluorenyl-2,7-diyl)-co-(1,4-{benzo-[2,1’,3]thiadiazole})]
FF: Fill factor
FIB: Focused ion beam
FRET: Fluorescence resonance energy transfer
FvdM: Frank-van der Merwe mode
FWHM: Full width at half maximum
G: Green
HAADF: High-angle annular dark field
HDA: Hexadecylamine
HOMO: Highest occupied molecular orbital
HPA: Hexyl-phosphonic acid
HRTEM: High resolution transmission electron microscopic
HTL: Hole transport layer
I_p_: Ionization potential
IR: Infrared
IR: Infrared
I_SC_: Short-circuit current
ITO: Indium tin oxide
LA: Lauric acid
LCAO: Linear combination of atomic orbital
LE: Luminous efficiency
LED: Light emitting devices
L_max_: Maximum luminance value
LUMO: Lowest unoccupied molecular orbital
MA: Methacrylic acid
MBE: Molecular beam epitaxy
MBE: Molecular beam epitaxy
MBP: Maltose binding protein
MDMO-PPV: Poly[2-methoxy-5-(3’,7’-dimethyloctyloxy)-1,4-phenylene vinylene]
Me: Methyl
MEHPPV: Poly[2-methoxy-5-(2-ethylhexyloxy)-1,4-phenylenevinylene]
ML: Monolayer
MMA: Methylmethacrylate
MO: Molecular orbital
MPA: Marcaptopropionic acid
MR: Magnetic resonance
MRI: Magnetic resonance imaging
MWNT: Multi-wall carbon nanotubes
NIR: Near infrared
NMR: nuclear magnetic resonance
O: Orange
OA: Oleic acid
OD: Optical density
ODA: Octadecylamine
ODE: 1-octadecene
OLED: Organic light emitting diode
OMeTAD: 2,2’,7,7’-tetrakis(N,N-di-p-methoxyphenyl-amine)9,9’-spirobifluorene
P3HT: Poly-3(hexylthiophene)
PCBM: [6,6]-phenyl C61 butyric acid methyl ester
PE: Power efficiency
PEDOT: Poly~3,4-ethylenedioxythiophene
PEDOT:PSS: Poly(3,4-ethylene-dioxy-thiophene) : poly(styrene-sulfonate)
PEG: Polyethylene glycol
PFBD: Poly(9,9’-dioctylfluorene-co-N-(4-butylphenyl)diphenylamine
PFCB: Perfluorocyclobutane
PL: Photoluminescence
PLE: Photoluminescence excitation
PP: Sodium polyphosphate
PPV: Poly(phenylene vinylene)
PPV: Poly(phenylene vinylene)
PS: Polystyrene
PSS: Polystyrene sulfonate
PVA: Poly(vinylalcohol)
PVB: Poly(vinybutryral)
PVD: Physical vapor deposition
PVK: Poly(vinyl-carbazole)
PVK: Polyvinyl carbazole
Qdot: Quantum dot
QLED: Quantum dots-based light emitting diodes
QW: Quantum well
QWQD: Quantum well quantum dot
QY: Quantum yield or quantum yield
R: Red
RD: Rhodamine
RIE: Reactive ion etching
SA: Stearic acid
SDS: Sodium dodecyl sulphate
SERS: Surface enhanced Raman spectroscopy
SK: Stranski-Krastonow mode
SPR: Surface plasmon resonance
STEM: Scanning transmission electron microscopy
SWNT: Single-wall carbon nanotubes
TAZ: 3-(4-Biphenylyl)- 4-phenyl-5-tert-butylphenyl-1, 2, 4-triazole
TBP: Tri-n-butyl phosphine
t-Bu-PBD: 2-(4-biphenylyl)-5-(4-tert-butylphenyl)-1,3,4 oxadiazole
TDPA: Tetradecylphosphonic acid
Tech-TOPO: Technical grade tri-n-octyl-phophine oxide
TEM: Transmission electron microscopy
TFB: Poly[(9,9-dioctylfluorenyl-2,70diyl)-co-(4-4’-(N-(4-sec-butylphenyl)) diphenylamine)]
TMS: trimethyl-silyl
TOA: trioctyl amine
TOP: Tri-n-octyl-phosphine
TOPO: Tri-n-octyl phosphene oxide
TPBI: 1,3,5-tris(N-phenylbenzimidazole-2-yl)-benzene
TPD: N, N’-diphenyl-N, N’-bis(3-methylphenyl)-(1, 1’-biphenyl)-4, 4’-diamine
UV: Ultraviolet
VM: Volmer-Weber mode
V_OC_: Open circuit voltage
vol.: By volume
V_turn-on_: Turn-on voltage of the device
W: White
XAFS: X-ray absorption fine structure
XPS: X-ray photoelectron spectroscopy
XRD: X-ray diffraction (XRD)
Y: Yellow
αNPD: 4,4-bis[N-(1-naphyl)-N-phenylamino]biphenyl
